# Xerophilic fungi contaminating historically valuable easel paintings from Slovenia

**DOI:** 10.3389/fmicb.2023.1258670

**Published:** 2023-11-02

**Authors:** Polona Zalar, Daša Graf Hriberšek, Cene Gostinčar, Martin Breskvar, Sašo Džeroski, Mojca Matul, Monika Novak Babič, Jerneja Čremožnik Zupančič, Amela Kujović, Nina Gunde-Cimerman, Katja Kavkler

**Affiliations:** ^1^Chair of Molecular Genetics and Biology of Microorganisms, Department of Biology, Biotechnical Faculty, University of Ljubljana, Ljubljana, Slovenia; ^2^Department of Knowledge Technologies, Jožef Stefan Institute, Ljubljana, Slovenia; ^3^Jožef Stefan International Postgraduate School, Ljubljana, Slovenia; ^4^Institute for the Protection of Cultural Heritage of Slovenia, Ljubljana, Slovenia

**Keywords:** fungi, easel paintings, contamination, damage, machine learning, biodeterioration, xerophiles

## Abstract

Historically valuable canvas paintings are often exposed to conditions enabling microbial deterioration. Painting materials, mainly of organic origin, in combination with high humidity and other environmental conditions, favor microbial metabolism and growth. These preconditions are often present during exhibitions or storage in old buildings, such as churches and castles, and also in museum storage depositories. The accumulated dust serves as an inoculum for both indoor and outdoor fungi. In our study, we present the results on cultivable fungi isolated from 24 canvas paintings, mainly exhibited in Slovenian sacral buildings, dating from the 16^th^ to 21^st^ centuries. Fungi were isolated from the front and back of damaged and undamaged surfaces of the paintings using culture media with high- and low-water activity. A total of 465 isolates were identified using current taxonomic DNA markers and assigned to 37 genera and 98 species. The most abundant genus was *Aspergillus*, represented by 32 species, of which 9 xerophilic species are for the first time mentioned in contaminated paintings. In addition to the most abundant xerophilic *A. vitricola*, *A. destruens*, *A. tardicrescens*, and *A. magnivesiculatus*, xerophilic *Wallemia muriae* and *W. canadensis*, xerotolerant *Penicillium chrysogenum*, *P. brevicompactum*, *P. corylophilum*, and xerotolerant *Cladosporium* species were most frequent. When machine learning methods were used to predict the relationship between fungal contamination, damage to the painting, and the type of material present, proteins were identified as one of the most important factors and cracked paint was identified as a hotspot for fungal growth. *Aspergillus* species colonize paintings regardless of materials, while *Wallemia* spp. can be associated with animal fat. Culture media with low-water activity are suggested in such inventories to isolate and obtain an overview of fungi that are actively contaminating paintings stored indoors at low relative humidity.

## 1. Introduction

Easel paintings represent the most diverse organic substrates in cultural heritage studies, therefore providing many different ecological niches that may be inhabited by various microbial species, including fungi (Ciferri, 1999). Fungal deterioration of cultural objects is increasingly studied to understand the mechanisms of fungal growth (Ciferri, 1999; Savković et al., 2019), consequent changes caused to the materials (Petersen and Klocke, 2013), and the best methods for restoration and preservation (Cappitelli et al., 2004). In comparison with wall paintings (Ciferri, 1999), canvas paintings are less studied. The literature is mainly limited to case studies (Santos et al., 2009; López-Miras et al. 2013a,b; Okpalanozie et al., 2016; Caselli et al., 2018; Calderón-Mesén et al., 2023), a few studies deal with collections (Paner, 2009; Vukojević and Grbić, 2010; Sabatini et al., 2018), and some reviews are available (Ciferri, 1999; Sterflinger, 2010; Petersen and Klocke, 2013; Poyatos et al., 2018; Poyatos-Jiménez et al., 2021; Savković et al., 2021).

Organic materials providing the necessary carbon source for fungal growth are present in the painting support (linen, hemp, jute, cotton, viscose, and polyester), as binders for the pigment egg white and yolk (albumin, ovalbumin, vitellogenin, lipids, casein, vegetable oils, vegetable gums, and starch), for the ground layer (animal glue such as collagen), and for the varnish layer (egg white, vegetable oils, natural resins, synthetic resins, vegetable gums, starch, and waxes). In addition, many conservation materials are of organic origin as well, such as waxes, animal glues (collagen), hydroxypropyl cellulose, and synthetic resins. As described by Paner (2009), all painting materials, except varnish, can serve as a nutrient source for saprophytic fungi. The necessary nitrogen source can be present in the support (keratin in wool, fibroin in silk), in the glues (e.g., collagen-based glues), or in emulsifiers/protectants (e.g., caseins in milk). Fungal growth was confirmed on dammar resin (Romero-Noguera et al., 2010) and shellac (López-Miras et al., 2013a), both commonly used in conservation-restoration. In contrast to the organic components, some pigments, mainly of inorganic origin, have an inhibitory influence on microbial growth (Paner, 2009).

The colonization of paintings by microbes usually occurs via dust deposits (Ciferri, 1999; Sterflinger, 2010; López-Miras et al. 2013a,b). Studies of airborne mycobiota in depositories and exhibition rooms correlated well with the fungal communities observed on the surface of paintings. The higher density and diversity of fungal communities on the front of the painting were explained as a direct consequence of the greater number of airborne fungal spores deposited on the surface of the painting (López-Miras et al., 2013a). Selected groups of heterotrophic microorganisms prevailed (Ciferri, 1999), characterized by high enzymatic activity and low nutritional requirements (Ciferri, 1999; López-Miras et al., 2013a). Most of the filamentous fungi associated with the damage of oil paintings on canvas were cellulolytically active and able to dissolve cellulose fibers, discolor the support, dissolve glues, or degrade oil binders (Sterflinger and Piñar, 2013).

The most commonly detected fungi on paintings were representatives of the saprotrophic genera *Aspergillus*, *Penicillium*, *Cladosporium*, and *Alternaria* (Caselli et al., 2018). *Aspergillus* and *Penicillium* species produce a variety of enzymes and organic acids (Savković et al., 2019) as well as mycotoxins (Frisvad, 2015); therefore, their presence poses a threat to visitors and restorers-conservators working with moldy materials (Skóra et al., 2015; de Carvalho et al., 2018). Due to their enormous enzymatic potential and ability to grow in low-water activity (a_w_) environments, fungi can colonize and decay paintings preserved even in relatively dry sites (Sterflinger, 2010). Fungal growth may cause aesthetic and structural damage, such as pigment discoloration, stains, deposits of visible mycelium, and degradation of support polymers, glues, and binders, resulting in the detachment of the paint layer from the support (Ciferri, 1999). Structural damage affects the aesthetic value and thus reduces the historical value of the painting. Restoration costs, in addition to the work of the conservator-restorer, also encompass expenses for microbiological analysis and disinfection. The cost of disinfection, removal of fungi, and retouching of the affected areas can vary from a few thousand to several tens of thousands of Euros, depending on the extent of the contamination and the size of the painting. However, originality and artistic value are irreparably changed and cannot be assessed by monetary value alone.

The aim of this study was to present the summarized results of a comprehensive examination of 24 canvas paintings exhibited in Slovenian churches and museums. They were either visibly infested with fungi on the front or back sides or heavily covered with dust containing fungal spores with biodeterioration potential. In addition to describing the extent of damage visible to the naked eye, we determined the material composition of the paintings. We isolated and identified the fungi that contaminated the paintings, with the goal of determining the usually overlooked xerophilic fungal communities that have deterioration potential at low relative humidity (RH). The data obtained were analyzed using machine learning methods that built predictive models for the occurrence of specific fungal taxa with painting materials or the damage caused. The results narrowed the list of fungal species that should be of particular importance in further studies on the restoration and conservation of canvas paintings.

## 2. Materials and methods

### 2.1. Measurements of environmental parameters

Temperature (T) and relative humidity (RH) were recorded by data loggers (Telehum) in the depository of the Institute for the Protection of Cultural Heritage of Slovenia, where all paintings assigned as RCS-were deposited prior to restoration. The average values for T and RH were 17.3 ± 3.1°C and 63.9 ± 8.5%, respectively, and overall, both parameters were within recommended values.

### 2.2. Selection of paintings

The majority of paintings were sampled once, the first in 2012 and the last in 2020. Three paintings were sampled more than once because they remained untreated in the depository during the time of our investigations. Fifteen paintings originated from Slovenian churches, two from a monastery in Slovenia, three from a private collection transported to Slovenia from France, and four from galleries in Slovenia. All paintings came from different locations with unknown long-term environmental conditions. However, 10 paintings from churches and 2 from a monastery were removed from their original locations and stored in a depository at the Institute for the Protection of Cultural Heritage of Slovenia (IPCHS) in Ljubljana for 2 or up to 10 years before our sampling.

### 2.3. Determination of painting materials

Wherever possible, at least two samples (paint chips) were taken from analyzed paintings, mainly from damaged areas or edges, for material analyses. The samples and documentation are stored in the IPCHS database.

A part of each extracted sample was embedded in polyester resin to prepare cross-sections for optical and scanning electron microscopy (SEM) as well as Raman spectroscopy, and a part was left unembedded for Fourier transform infrared (FTIR) spectroscopy.

FTIR spectrometry analyses were carried out in transmission mode after sample chips were compressed in a diamond anvil cell, enabling analyses of all present layers. Spectra were scanned in the range of 4,000 cm^−1^ to 600 cm^−1^ at a resolution of 4 cm^−1^, averaging 32 scans into a single spectrum. Optical microscopy was carried out on embedded cross-sections with an Olympus BX60 microscope at 200× and 500× magnifications. Raman spectra were scanned with a LabRAM HR800 spectrometer (Horiba Scientific, France) connected to an Olympus BXFM (Japan) microscope by using a laser of 785 nm wavelength. A CCD detector with a spectral resolution of approximately 1 cm^−1^ was used. Spectra were scanned in the range between 100 cm^−1^ and 2,000 cm^−1^ (or 2,500 cm^−1^ for blue pigments). The elemental composition of the paint samples, which did not give a definitive result by Raman spectroscopy, was additionally analyzed by a scanning electron microscope (SEM) JEOL 5500 LV (Japan) with an energy dispersive spectrometer (EDS) OXFORD in low vacuum mode. Canvas fibers were analyzed by the same microscope in transmission mode, prepared as temporary preparations, i.e., single fibers in distilled water.

### 2.4. Selection and characterization of sampling sites and on-site visualization of fungal contaminations

Contamination of the paintings was visible to the naked eye in most cases and was confirmed in some paintings by USB microscopy at the sampling site, and in the majority of paintings also by taking adhesive tape samples (Urzi and de Leo, 2001) and subsequent microscopy in the laboratory using the BX51 Olympus microscope. Photomicrographs of adhesive tape samples were taken with a DP72 camera (Olympus). Microscopy of adhesive tape samples allowed for genus-level microscopical identification of common colonizers on the paintings on the basis of morphological characters (e.g., *Alternaria*, *Aspergillus*, *Penicillium*, *Cladosporium*, and *Chaetomium*). Sometimes only mycelium without conidia or supporting structures was observed, or conidia were observed that could not be assigned to a particular genus. Damage to the paintings was recorded on the basis of paint layer/support discoloration (white, gray, brown, and green), visible cracking or peeling paint layers, mycelial growth in the cracks, and torn canvas.

### 2.5. Isolation of fungi from the sampled paintings

#### 2.5.1. Sampling

Samples were collected from areas displaying clear evidence of fungal colonization in a non-invasive manner using swabs moistened with sterile 0.9% (w/v) NaCl solution. Additionally, Copan ESwabTM (collection and transport system, Copan Diagnostics Inc.) was also utilized for samples collected in 2019 and 2020. Furthermore, paintings AIO-1, AIO-2, AIO-3, and RCS15 were also directly imprinted onto DG18 medium-filled RODAC plates (replicate organism detection and counting plates). In cases of compact and localized overgrowths, small areas of approximately 1 cm^2^ were sampled, whereas areas of 100 cm^2^ were sampled for larger, scattered contaminations. Each area was sampled in duplicate (two cotton swabs per sample), and the cotton swabs were stored in sterile dry tubes at 4°C and then processed within 24 h. Both replicates were equally inoculated onto different culture media (specified below) by swabbing the entire surface uniformly. In cases where Copan ESwabs were also used (one ESwab per sample), after sample collection, they were placed into sterile transport Amies liquid, stored at 4°C, and processed within 24 h. The ESwab tubes were vigorously shaken to release the inoculum from the brush into the liquid. Subsequently, the brush was removed, and 100 μL of this suspension was inoculated and evenly spread over the surface of various culture media, as was indicated previously for the cotton swabs.

#### 2.5.2. Culture media

The following solid culture media, all supplemented with chloramphenicol (50 mg/L) and characterized by different water activities (a_w_), were used for all the isolations: Dichloran Rose Bengal Chloramphenicol agar (DRBC; Merck, Germany; a_w_ 0.997), Dichloran Glycerol agar (DG18; Oxoid, England; a_w_ 0.955), and malt-yeast agar with 50% glucose (MY50G; a_w_ 0.89; Pitt and Hocking, 2009). Paintings sampled in the year 2012 (RCS 15–RCS 26) were processed on additional culture media: Malt Extract Agar (MEA; a_w_ 0.99; Pitt and Hocking, 2009), MEA with the addition of 5% NaCl (MEA5% NaCl; a_w_ 0.965; Gunde-Cimerman et al., 2000), malt-yeast agar with 10% NaCl and 12% glucose (MY10-12; a_w_ 0.88; Pitt and Hocking, 2009), and also on bacteriological media including nutrient agar (NA) supplemented with 5% NaCl and M9 Minimal Medium (M9, Elbing and Brent, 2002), both media used without and with the supplement of cycloheximide.

#### 2.5.3. Incubation conditions, pure culture isolation, and storage

All inoculated plates were incubated at 25°C for up to 4 weeks. Representatives of morphologically similar operational taxonomic units were transferred to fresh solid culture media: those from high-water activity media (DRBC, MEA) were subcultured on MEA and those originating from low a_w_ media on DG18. Representative strains of encountered operational taxonomic units were qualitatively assessed. Isolated pure cultures were deposited in the Ex Culture Collection of the Infrastructural Centre Mycosmo, MRIC UL, Slovenia,^1^ at the Department of Biology, Biotechnical Faculty, University of Ljubljana.

### 2.6. Identification of fungi

The majority of isolated fungi were assigned to a genus based on their macroscopic culture characteristics and conidiophore and spore morphology assessed with a stereo microscope (Olympus SZ61) or the earlier specified Olympus light microscope. Pure cultures were identified by DNA barcodes suitable for morphologically identified, individual genera. The genomic DNA of yeast isolates was extracted using PrepMan Ultra reagent (Applied Biosystems) according to the manufacturer’s instructions. DNA from filamentous fungi was extracted after mechanical lysis in CTAB buffer according to the protocol described by Gerrits van den Ende and de Hoog (1999). Identification to species level was performed by sequencing genus-specific recommended molecular taxonomic marker loci: beta-tubulin (benA) for *Aspergillus* and *Penicillium*, actin (Act) or elongation factor (TEF1α) for *Cladosporium*, internal transcribed spacers 1 and 2 including the 5.8S rDNA (ITS) for all other fungi. These were amplified and sequenced using the following primer sets: Ben2f (Hubka and Kolarik, 2012)/Bt2b (Glass and Donaldson, 1995), ACT-512F/ACT-738R (Carbone and Kohn, 1999), EF1-983F/EF1-2218R (Rehner and Buckley, 2005), and ITS1/ITS4 (White et al., 1990). Following sequencing, we conducted similarity searches by querying the sequences against the National Center for Biotechnology Information (NCBI) nucleotide database using the BLASTn search algorithm. The conspecificity of the analyzed isolates was tested in preliminary phylogenetic analyses using the maximum likelihood approach as implemented in Mega7 (Kumar et al., 2016).

All DNA sequences of the representative strains from this study were deposited in the GenBank database: MW288060–MW288072, MW288703–MW288753, MW288758–MW288770, MW288930–MW288938, MW289524–MW289546, OQ410476–OQ410483, OR097691 (ITS rRNA for the majority of isolates), MW369716–MW369733, MW387131–MW387142, OR102450–OR102458 (actin for *Cladosporium* isolates), MW387143–MW387152 (elongation factor for the *Cladosporium* isolates), MW357077–MW357266, MW369686–MW369715, OQ420406–OQ420423, OQ446663–OQ446691, and OR102446–OR102449 (beta-tubulin for the *Aspergillus* and *Penicillium* isolates).

### 2.7. Statistical analysis of results

To investigate potential connections between the sampling locations, painting techniques, and the presence of certain fungal contaminants on paintings, the data were classified and visualized in the environment R (R Core Team, 2022) and Microsoft Excel 2016.

Hierarchical clustering was employed to determine the similarity among samples using the “hclust()” function. Principal component analysis (PCA) was performed with the “prcomp()” function. Both functions are from the package “stats” v3.6.1, which is part of base R.

### 2.8. Machine learning

In machine learning (ML), the task of binary classification is addressed by training models to predict the presence/absence of a specific target. Our analysis scenarios are more general since we are interested in predicting the presence/absence of several fungi. Hence, the machine learning task we have to address is called multi-label classification (MLC). With MLC, each label corresponds to a specific fungus. A particular sample in the dataset can be annotated with more than one label. Generally, a MLC task can be addressed locally by learning one model for each label or globally by learning one model for all labels together. Depending on the domain, the targets themselves can be part of a hierarchy, e.g., kingdoms, orders, families, genera, sections, and species. In such cases, the hierarchy can be used as an input into the learning algorithm, and the ML task is called hierarchical MLC (HMLC). We will be using predictive clustering trees (PCTs) (Blockeel and De Raedt, 1998; Vens et al., 2008), which are capable of globally solving (H)MLC tasks.

If one wants to make a prediction with a PCT, the procedure is as follows: A single PCT (as with any other decision tree) starts with a root node. All nodes, with the exception of bottom-most nodes (i.e., leaf nodes), contain a single attribute name. At the beginning of the prediction process, a sample is passed into the root (topmost) node. The sample’s value of the said attribute is used to determine the next node. The sample is then passed down the tree until it reaches a leaf node. Leaf nodes contain predictions. In our analysis, the predictions are labels (targets), representing fungi that are, according to the model, present in the observed sample.

Any decision tree can be used in a descriptive and/or predictive manner. The predictions can be explained by the path (from the root to the leaf node) that a sample had to travel. In cases where the model is not suitable for making predictions, a PCT can still be used as a clustering model (descriptions of clusters are paths from root to leaf nodes, as explained above).

We also use a feature importance estimation technique based on random forest ensembles of PCTs (Petković et al., 2020). The result of such analysis is a list of attributes that are present in the observed dataset, ordered by their importance with respect to the learning target(s). The ranking scores are internal to the learning algorithm and are not comparable between experiments. However, they are informative within a single experiment, i.e., an attribute with a ranking score of *X* is twice as important as an attribute with a ranking score of $\frac{X}{2}$.

Each analysis scenario has its own set of inputs and outputs (labels). For every scenario, PCTs were trained using all available input features and were allowed to grow until all leaf nodes contained at most two examples (pre-pruning). The quality of the trained predictive models was evaluated by calculating the weighted area under the precision-recall curve (AUPRC). Since no separate test was available, the predictive performance was estimated using 10-fold cross-validation. Feature importance was calculated using random forest ensembles with 100 PCTs, using 75% of the input features for random selection and the Genie3 scorer.

All methods are implemented within the CLUS software, available at https://github.com/knowledge-technologies/clus.

## 3. Results

### 3.1. Determination of painting materials

**Figure 1:**
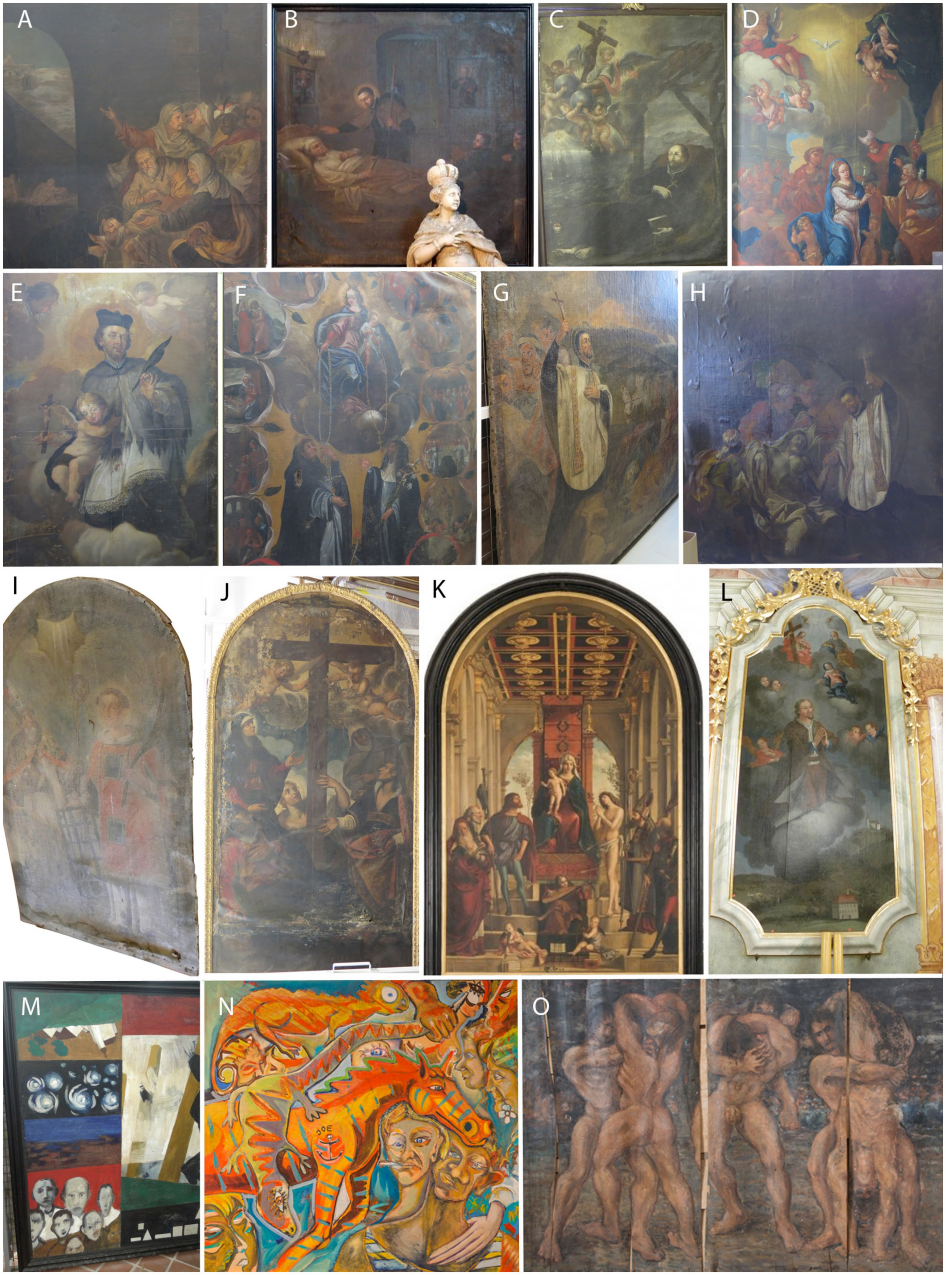
Representative sampled paintings. **(A)** RCS 15; **(B)** RCS 16; **(C)** RCS 17; **(D)** RCS 20; **(E)** RCS 21; **(F)** RCS 22; **(G)** RCS 25; **(H)** RCS 26; **(I)** ART-4; **(J)** RCS 24; **(K)** ART-1; **(L)** ART-5; **(M)** GBJ-1; and **(N)** GM-1 (Courtesy of Katarina Brešan); **(O)** GM-2 (Courtesy of Katarina Brešan). Painting codes are explained in Supplementary Table S1.

Most of the paintings examined (Figure 1) were painted on canvas (flax, hemp, or unknown), one on hardboard, and one on veneer. Most of the paintings (17) were painted with oil paints; in four, we were unable to identify the pigment binder, as it was either oil or greasy tempera. One painting was painted with acrylic paint, one with oil and greasy tempera, and one with greasy tempera only (Supplementary Table S1). Wax was identified as a consolidant or lining material in seven paintings. Natural resins were identified in four paintings, always in varnish layers. Proteins were identified in six paintings, either in the ground layers, in the canvas isolation, or, in one case, as a consolidant of the paint layer during conservation-restoration. Starch was identified as a component of one painting.

### 3.2. Observed damage on moldy paintings

**Figure 2:**
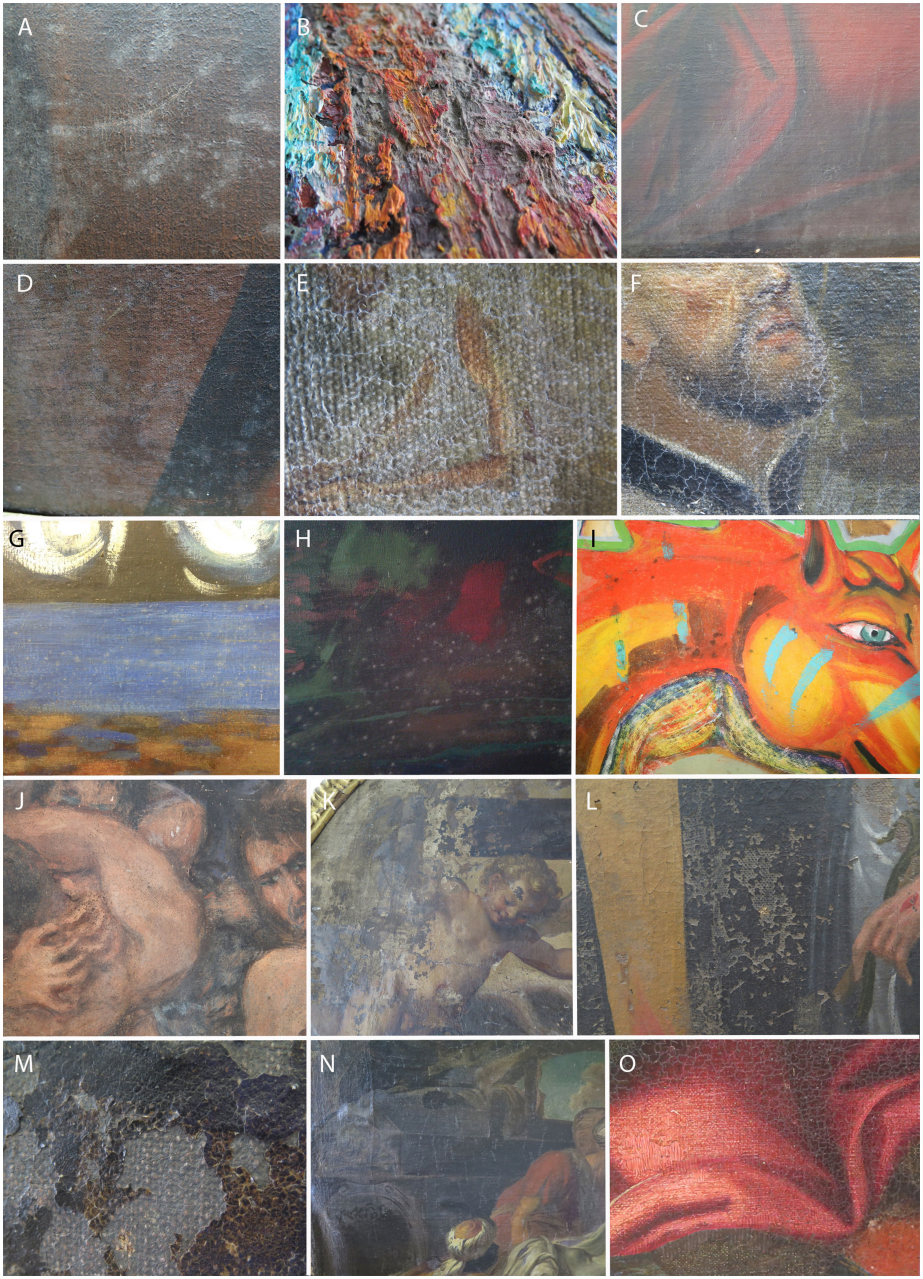
Documented damages on the obverse sides of the sampled paintings, potentially caused by fungi. **(A)** RCS 20; **(B)** AIO-1; **(C)** ART-1; **(D)** RCS 20; **(E,F)** RCS 25; **(G)** GBJ-1; **(H)** GBJ-1; **(I)** GM-1; **(J)** GM-1; **(K)** RCS 24; **(L)** RCS 22; **(M,N)** RCS 26; and **(O)** ART-2. Painting codes are explained in Supplementary Table S1.

Mold growth was observed on the front of most paintings (Figure 2), particularly as light-colored mycelium on darkly pigmented painting surfaces (RCS 20; Figure 2A). On paintings with impasto-applied layers of paint, spider webs were associated with mold growth (AIO-2, 3; Figure 2B). A non-glossy (matte) surface was observed on otherwise glossy paintings due to either mold growth or dust accumulation (ART-1; Figure 2C). Many paintings exhibited white mycelium, either in the form of defined white patches (e.g., RCS 23; Figure 2D) or less visible mycelium threats (e.g., RCS 25; Figure 2E), the latter often originating from cracks in the paintings (RCS 25, 26; Figure 2F). Stains of varying coloration were observed on the front sides, associated with specific pigments in some paintings, e.g., blue (e.g., GBJ-1; Figure 2G), brown, and green (e.g., GBJ-2; Figure 2H). Sometimes the stains were dark (brown and black) and occurred on a specific pigment (e.g., GM-1, GM-2; Figures 2I,J). In some paintings, a change in paint layer consistency was observed, so that the paint layer appeared weathered (RCS 22) or even powdery (RCS 17). Mycelium was often associated with paint flaking (RCS 21; Figure 2K) or peeling (RCS 26; Figure 2L). In one case, a folded paint layer was noted (RCS 26; Figure 2M), and in a few cases, mechanical damage in the form of tearing (RCS 23; Figure 2N) or thinned canvas was noted (RCS 18). Discoloration was observed on the waxed surface (RCS 24; Figure 2O). Peeling and cracking were otherwise observed in areas of the painting that were not visually moldy (ART-2; Figure 2P). White stains, presumably of fungal origin, were also observed on a wooden frame (RCS 19).

**Figure 3:**
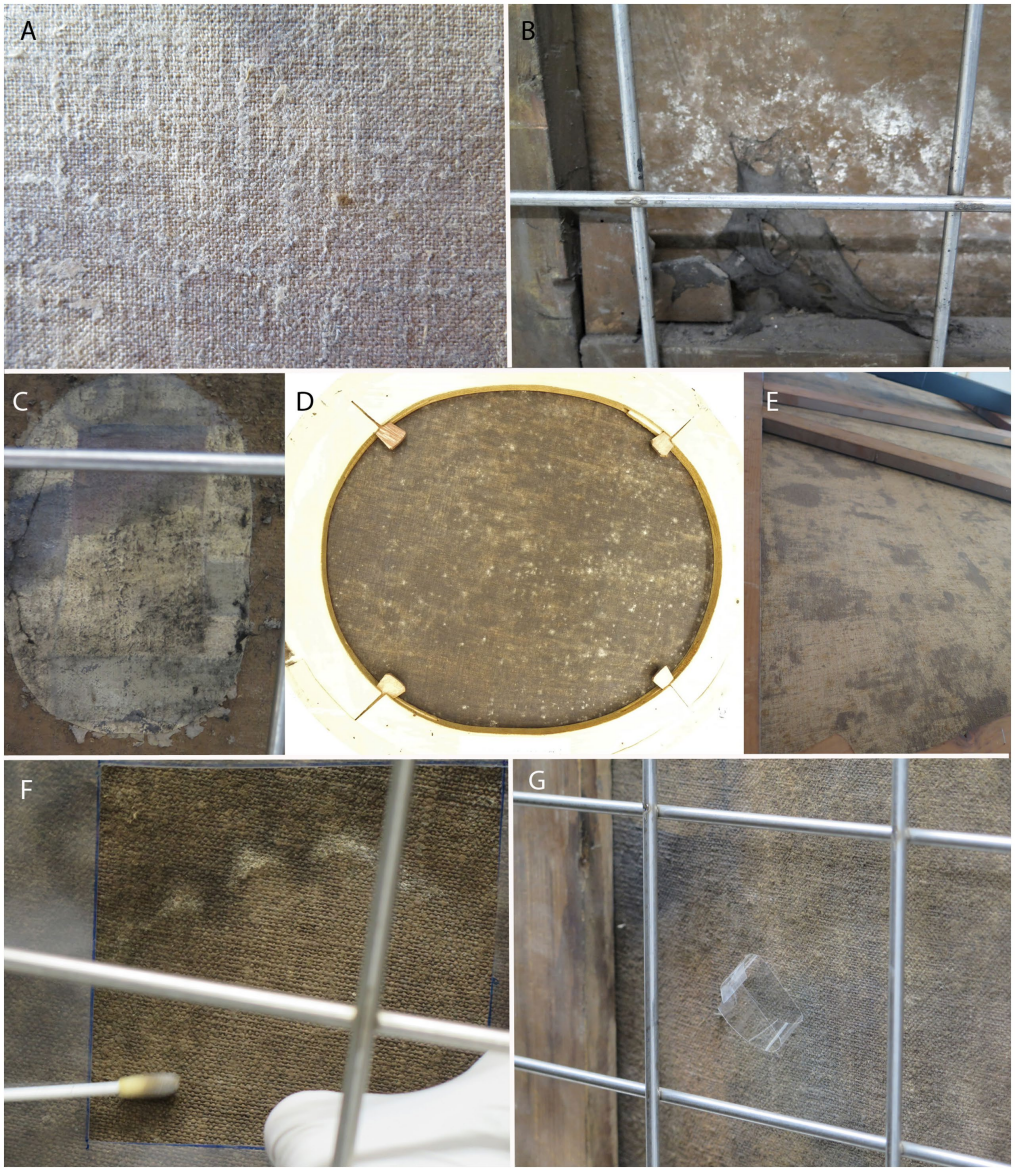
Appearance of the moldy reverse painting sides. **(A,E)** ART-1, **(B,C)** RCS 15, **(D)** RCS 36, **(F)** ART-22—sampling for fungi with a swab, and **(G)** ART-22—taking tape print samples. Painting codes are explained in Supplementary Table S1.

Fungal infestations on the back of paintings (Figure 3) were often observed in the form of stains (white, gray, and green) that were more evident on darker canvases (Figure 3D) and less evident on light, non-contrasting canvases (Figure 3A). Some paintings were covered with cobwebs (Figure 3B); mold was also observed on glued canvases (RCS 15; Figure 3C). In addition, penetration of the lining glue through the canvas often hindered the visible examination and detection of mold on the back of paintings.

### 3.3. *In situ* visualized fungi on paintings

**Figure 4:**
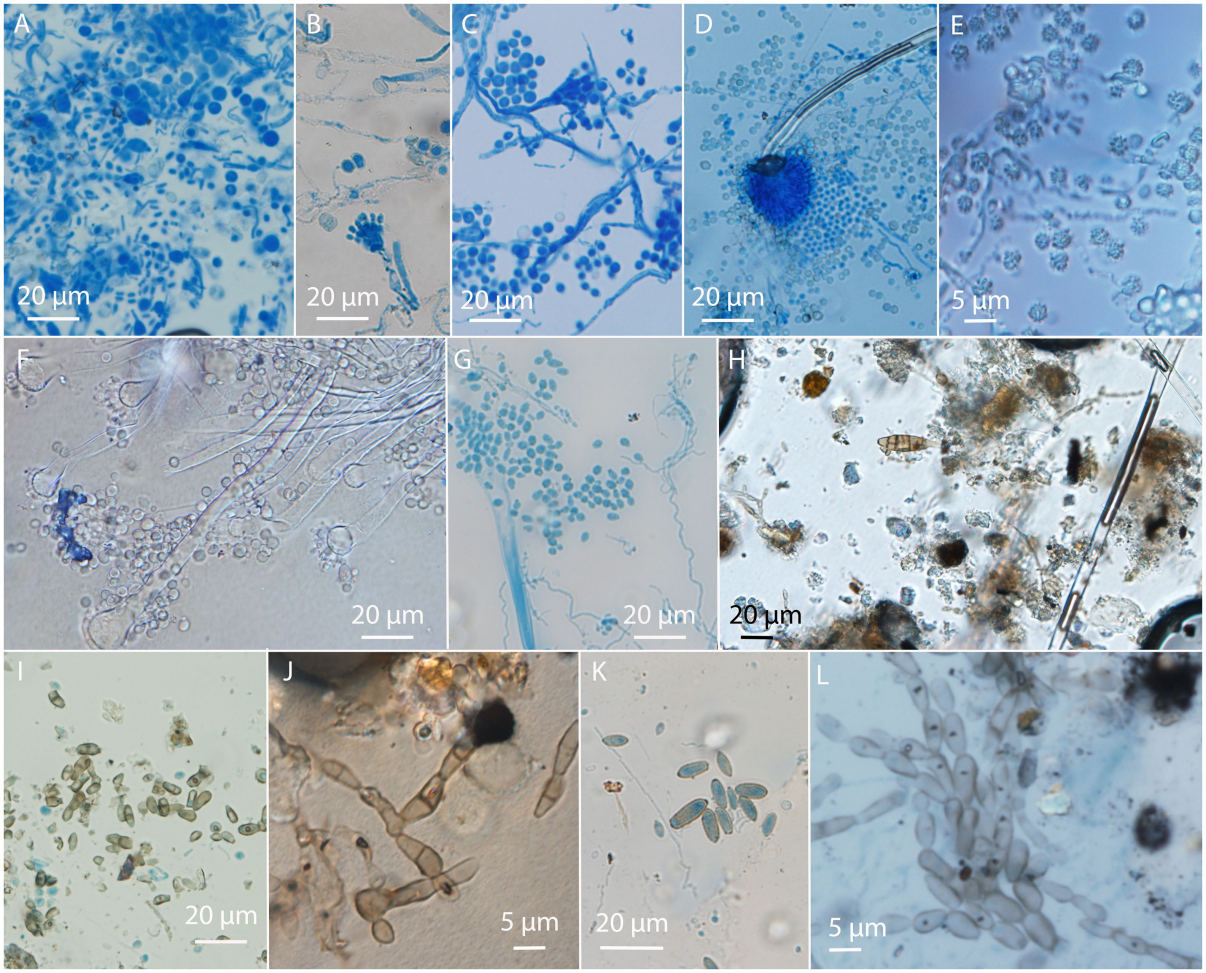
Visualization of fungal structures on adhesive tape prints taken from paintings observed by optical microscopy. *Aspergillus* on **(A–G)**; **(A,B)** GBJ-1, **(C)** GBJ-2, **(D,E)** AIO-2, **(D)** AIO-1, **(F)** ART-5, **(G)** RCS 26; *Alternaria* and *Cladosporium*-like conidia **(H–L)**; **(H)** ART-4, **(I)** RCS 22, **(J)** ART-4, **(K)** RCS 25, and **(L)** RCS 22. Painting codes are explained in Supplementary Table S1.

Using a USB microscope on-site helped us identify moldy areas of the paintings. In some of these areas, mycelium and sporulation structures were observed. More precise identification of fungi was possible by the microscopy of collected translucent tape samples to observe the conidiogenous apparatus, conidial morphology, and arrangement (Figure 4). The most frequently encountered were fungi belonging to the genus *Aspergillus*, observed on nine paintings based on the presence of typical conidial heads. On six paintings, *Cladosporium-* and/or *Cladosporium*-like taxa were recognized, while the *Penicillium* genus was recognized on two paintings (Supplementary Table S2).

### 3.4. Fungi isolated from paintings

Fungi were isolated from all 24 sampled paintings (Supplementary Tables S2, S3). Altogether, we obtained 465 fungal isolates and assigned them to 37 genera and 98 species. Of these, the most frequently encountered were the following genera: *Aspergillus*, represented by 31, *Penicillium* by 16, and *Cladosporium* by 10 species.

**Figure 5:**
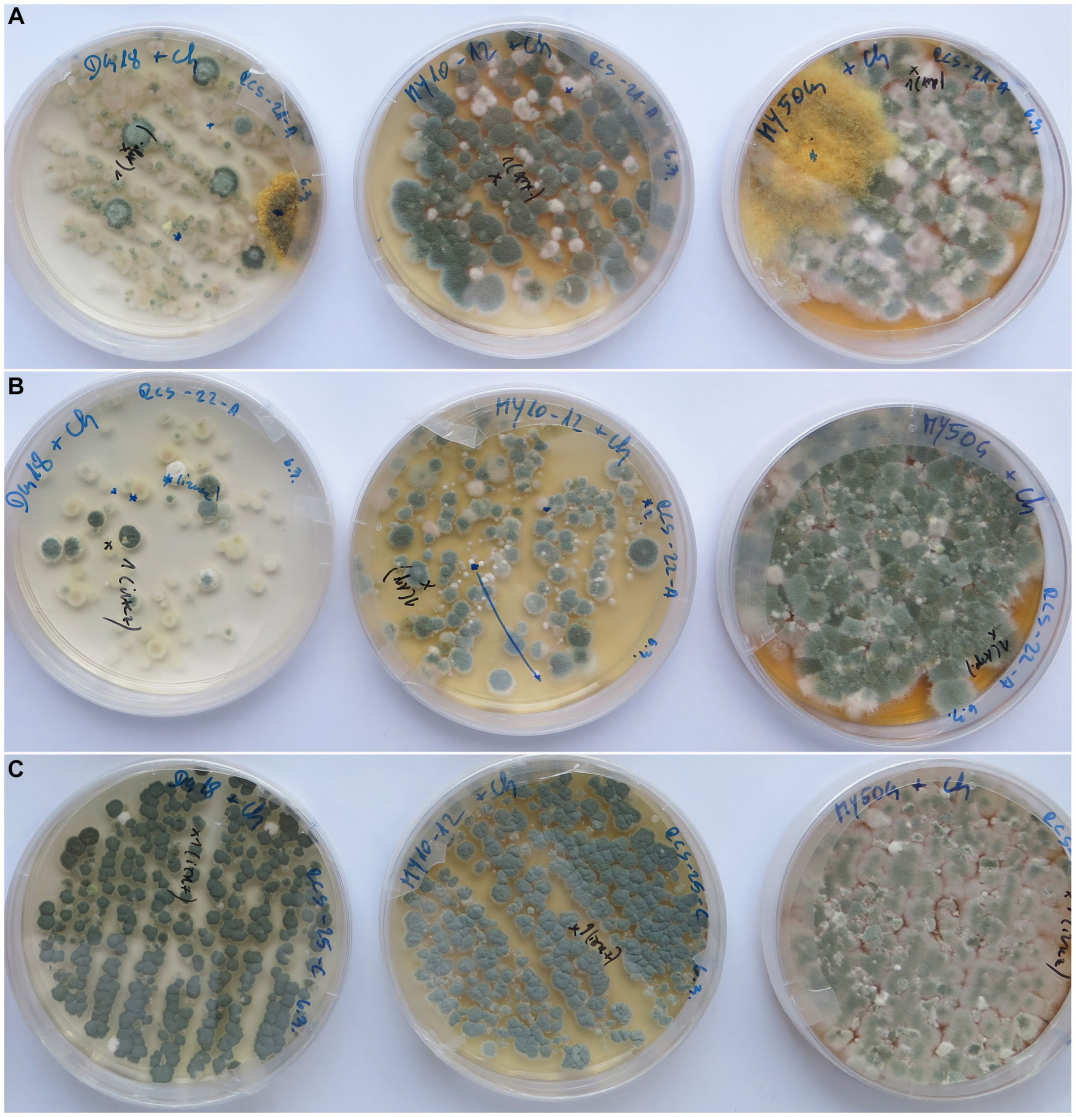
Low-water activity culture media with a variety of fungal colonies from deteriorated historic paintings (from left to right: DG18, MY10-12, and MY50G). **(A)** Painting RCS 21—front side, **(B)** painting RCS 22—front side, and **(C)** painting RCS 25—back side.

Swabs smeared on culture media resulted in much higher fungal CFU numbers (almost confluent growth) and isolated species if they were spread on low-water activity media, such as DG18, MY50G, and MY10-12; they yielded 46, 35, and 17 fungal species, respectively. High-water activity media, such as DRBC and MEA, contained only several colonies per plate and yielded 47, and 14 fungal species, respectively (Supplementary Tables S3, S4 and Figure 5). Culture media MEA + 5%NaCl, NA + 5%NaCl, and M9 were used only occasionally, and therefore fewer taxa were recorded on them (6, 3, 3, respectively) (Supplementary Table S4).

**Figure 6:**
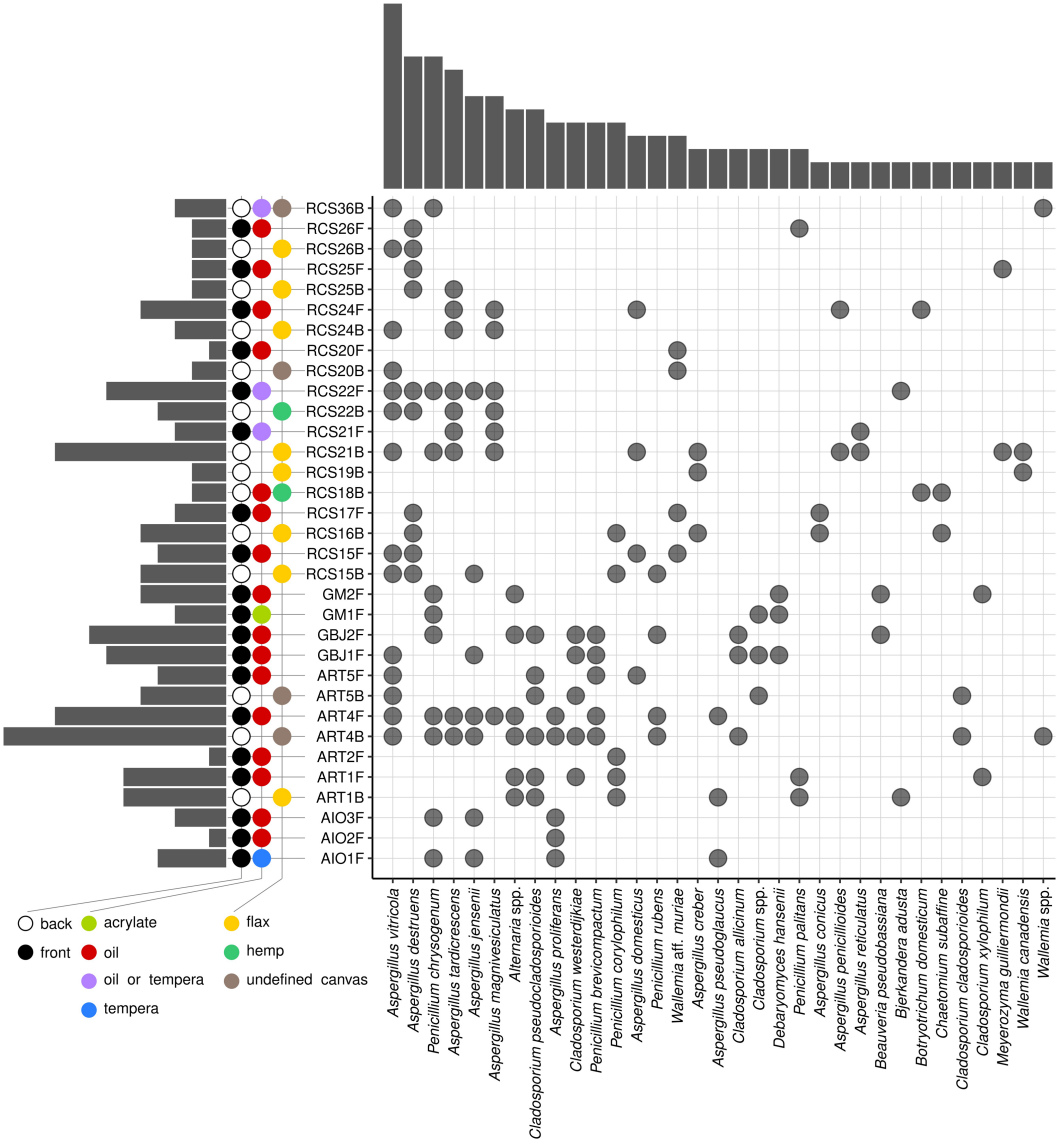
Upset diagram of the occurrence of fungal species on paintings. Only species occurring in at least two paintings are presented. Different supports, painting techniques, and painting sides are presented in color codes. Columns at the left side of the graph represent the number of isolated species per painting, and columns above the graph represent the number of paintings where certain species were isolated.

Fungal species present on at least two paintings belonged to 12 *Aspergillus*, 5 *Penicillium*, 6 *Cladosporium*, 3 *Wallemia*, and a single species of the genera *Alternaria*, *Debaryomyces*, *Beauveria*, *Bjerkandera*, *Botryotrichum*, *Chaetomium*, and *Meyerozyma* (Figure 6). Eight of twelve (67%) *Aspergillus* species belonged to *Aspergillus* section *Restricti* (*A. vitricola*, *A. destruens*, *A. tardicrescens*, *A. magnivesiculatus*, *A. domesticus*, *A. conicus*, *A. penicillioides*, and *A. reticulatus*), two (16.5%) to the *A.* section *Aspergillus* (*A. proliferans* and *A. pseudoglaucus*), and two (16.5%) to the *A.* section *Versicolor* (*A. versicolor* and *A. creber*). *Penicillium chrysogenum* was the most frequently isolated *Penicillium* species, in addition to *P. brevicompactum*, *P. corylophilum*, *P. rubens*, and *P. palitans* (Figure 6).

Of the 98 species isolated in total, 53 belonging to 26 different genera were found exclusively on one painting and were not identified in samples from any other paintings. These were *Akanthomyces muscarius*, *Arthrinium arundinis*, *Arthrinium marii*, *Aspergillus flavus*, *A. glaucus*, *A. halophilicus*, *A. infrequens*, *A. kumbius*, *A. luchuensis*, *A. montevidensis*, *A. neocarnoyi*, *A. niveoglaucus*, *A. oryzae*, *A. pragensis*, *A. ruber*, *A. salinicola*, *A. sloanii*, *A. steynii*, *A. sydowii*, *Botryotrichum murorum*, *Botrytis cinerea*, *Chaetomium cochliodes*, *Chaetomium* sp., *Cladosporium neolangeronii*, *Cl. perangustum*, *Cl. velox*, *Coniochaeta ligniaria*, *Coprinellus micaceus*, *Curvularia coatesiae*, *Cylindrobasidium* sp., *Dichotomopilus erectus*, *Dothiora* sp., *Filobasidium wieringae*, *Hypoxylon perforatum*, *Epicoccum* sp., *Neosetophoma guiyangensis*, *Penicillium bialowiezense*, *P. citrinum*, *P. expansum*, *P. glabrum*, *P. polonicum*, *P. raistrickii*, *P. scabrosum*, *P. steckii*, *P. tardochrysogenum*, *Peniophora cinerea*, *Periconia pseudobyssoides*, *Peroneutypa scoparia*, *Pseudopithomyces palmicola*, *Stereum hirsutum*, *Stereum* sp., *Talaromyces rugulosus*, *Trichoderma longibrachiatum*, and *Zalaria obscura.*

On average, most paintings exhibited between 4 and 5 different fungal species. Only one species was isolated in 2 of the 24 sampled paintings: *Aspergillus* sp. on RCS 23 and *A. proliferans* on AIO-2. The maximal number, 19 species, were cultured from the front side of the painting ART-1 and from the front and back sides of the painting ART-4. On control sampling sites, where we could not detect fungal contamination with the naked eye, some species, otherwise detected also on visually moldy sites, were encountered, such as *A. destruens* and *A. vitricola* on RCS 15, and *A. vitricola* on RCS 22, or other environmental fungi not isolated from moldy sites of paintings. Although not the focus of this study, a parallel investigation was conducted in which environmental samples (air and damp moldy walls of the RCS depository; data not shown) were collected. Some species, such as *A. creber*, *A. jensenii*, *A. pseudoglaucus*, *A. tardicrescens*, *A. vitricola*, *Debaryomyces hansenii*, and *Wallemia* aff. *muriae*, were identified in both the paintings and the environmental samples, suggesting a potential source of inoculum and/or cross-contamination to the paintings.

### 3.5. Statistical evaluation of results

Since most isolated fungi appeared only on individual paintings, statistical analyses were performed on the presence/absence of data for species occurring on at least two sampled paintings.

Among the isolates from paintings, we observed a statistically significant co-occurrence of the following pairs of species: *Alternaria* sp. and *Cladosporium pseudocladosporioides* (prob. co-occurrence = 0.033), *Aspergillus jensenii* and *A. proliferans* (*p* = 0.032), and *A. proliferans* and *P. chrysogenum* (*p* = 0.046).

Differences in the number of taxa isolated from the front and back sides of the paintings were not statistically significant (ANOVA *p* > 0.05).

**Figure 7:**
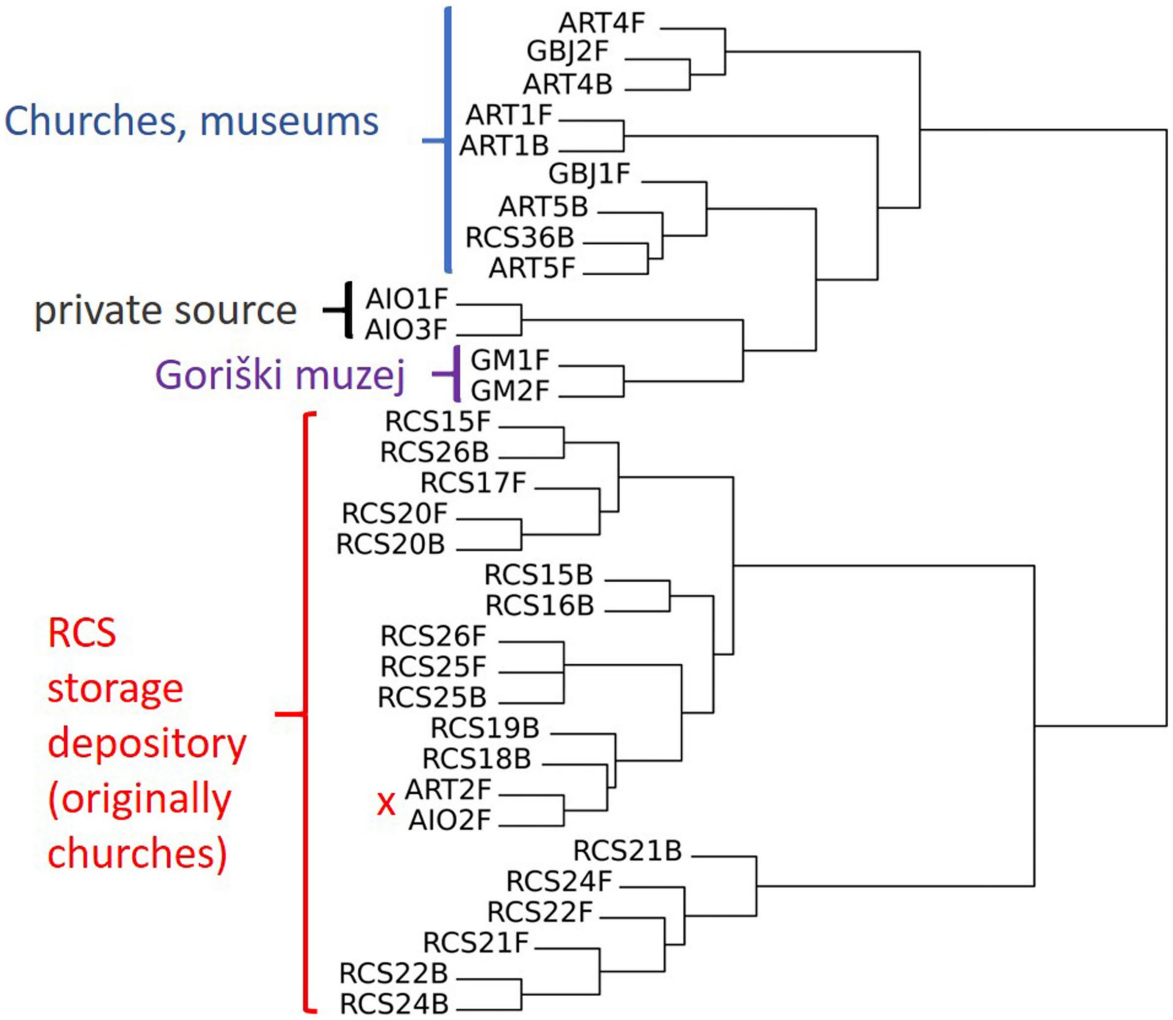
Hierarchical clustering analysis of the paintings based on the presence/absence of cultivable fungi, with additionally labeled sampling sides (F = front side; B = back side).

**Figure 8:**
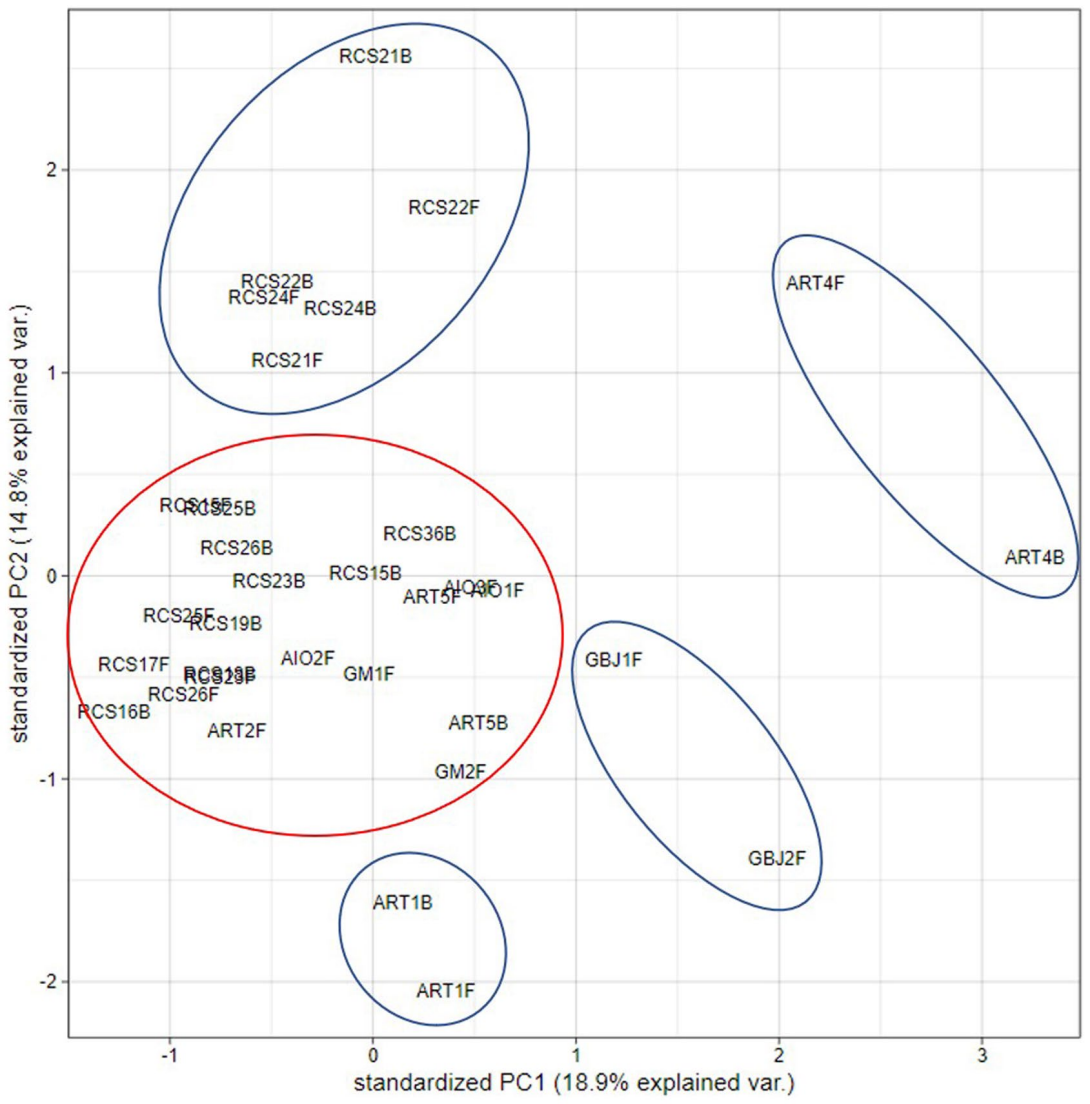
Principal component analysis (PCA) plot for the first two principal components, using the data on the presence/absence of isolated fungal species from the obverse and reverse sides of paintings.

In the hierarchical clustering analysis, we observed two main clusters. One cluster consists of 13 paintings that were kept for a longer period of time in the RC depository (Ljubljana) and 2 paintings that were kept and sampled in other locations, ART-2F (Izola, Slovenia) and AIO-2F (Corsica, France). The second cluster contains paintings that were sampled primarily at sites—churches, and those originating from a private collection. In this cluster, there are many sub-clusters, e.g., with two (out of three sampled) paintings from a private collection (AIO), two paintings from the Gorica museum (GM-1 and GM-2), and a cluster with two sampling sides (front and back) of the same painting from the church in Koper (ART-1). Two sub-clusters contain single paintings sampled from the front and back (ART-4 and ART-5); both clusters contain an additional painting from a gallery (GBJ-1 and GBJ-2). It is also evident that some paintings carry similar mycobiota on the front and back surfaces, as in the case of RCS 20, RCS 25, and ART-1 (Figure 7). PCA analysis shows similar results, and we observe clusters of paintings either from the same repository (RC) or from the same painting sampled on the front and back sides (Figure 8).

### 3.6. Determination of mold species on deteriorated parts of paintings

Species of the genus *Aspergillus* sect. *Restricti* were the most common fungi on damaged paintings in our study, based on the frequency of isolation and their *in situ* presence observed on tape prints (see Supplementary Table S2). *Aspergillus vitricola* was the only contaminating species on two drying oil paintings (RCS 20 and ART-5), while it coexisted with several other *Aspergillus* species on two other paintings (RCS 22, GBJ-1). *Aspergillus destruens* was isolated from two drying oil paintings on flax canvas (RCS 15 and RCS 26) and from two such paintings containing additional proteins (RCS 16 and RCS 17); it also contributed to a mixed *Aspergillus* infection of painting RCS 25, where it grew in paint cracks. The later phenomenon was also observed in RCS 26. *Aspergillus proliferans* (*A*. sect. *Aspergillus*) was detected on deteriorated parts of two 20th-century paintings (AIO-1 and AIO-2). Several *Aspergillus* species were found on Baroque drying oil paintings on canvas containing wax (RCS 21, RCS 24, and RCS 25), while some of such paintings (RCS 22, RCS 36, GBJ1, and GBJ2) also contained other fungi of the genera *Penicillium*, *Cladosporium*, *Wallemia*, and *Trichoderma*. *Cladosporium* was considered an active contaminant of a drying oil painting (ART-4). *Aspergillus jensenii* and *A. creber* were identified on the 20th-century drying oil painting containing wax (RCS 19) and were also isolated from other paintings with mixed infections. *Debaryomyces hansenii* was isolated from three paintings. We considered it to be the active contaminant of the front lower part of a 20th-century oil painting on veneer (GM-2), which showed visual damage in the form of small scattered black spots. Black yeast species, identified as *Zalaria obscura*, was found on patches of similar appearance in the upper part of the painting (GM-2); unfortunately, we have no direct microscopic data from this painting. Although we isolated several fungal colonies from the 16th-century drying oil paintings RSC 23, ART-1, and ART-2, we assume that these paintings do not contain actively growing fungi, as we did not observe mycelial growth on tape print samples from deteriorated areas. From one of these paintings (ART-1), we even observed and determined one of the most diverse fungal communities in this study—18 different species represented by individual colonies.

### 3.7. Prediction models derived from machine learning

From the data obtained on the materials, sampling sites, damages, visualized fungi from tape off-prints, and from the list of isolated species, we analyzed relations among different features using six scenarios. Each scenario consists of several input features and target(s). We analyzed the following scenarios: (i) inputs: sampling, culture media, targets; isolated fungi; (ii) inputs: material, sampling sites, targets: microscopically detected fungi; (iii) inputs: material, sampling sites, targets: isolated fungi; (iv) inputs: observed damages, targets: detected fungi; (v) inputs: observed damages on paintings, targets: cultured fungi; (vi) inputs: damages, targets: *Aspergillus* species. For each scenario, a model (PCT) and feature importance scores are provided.

#### 3.7.1. Painting material composition and sampling sites in relation to on-site detected fungi

**Figure 9:**
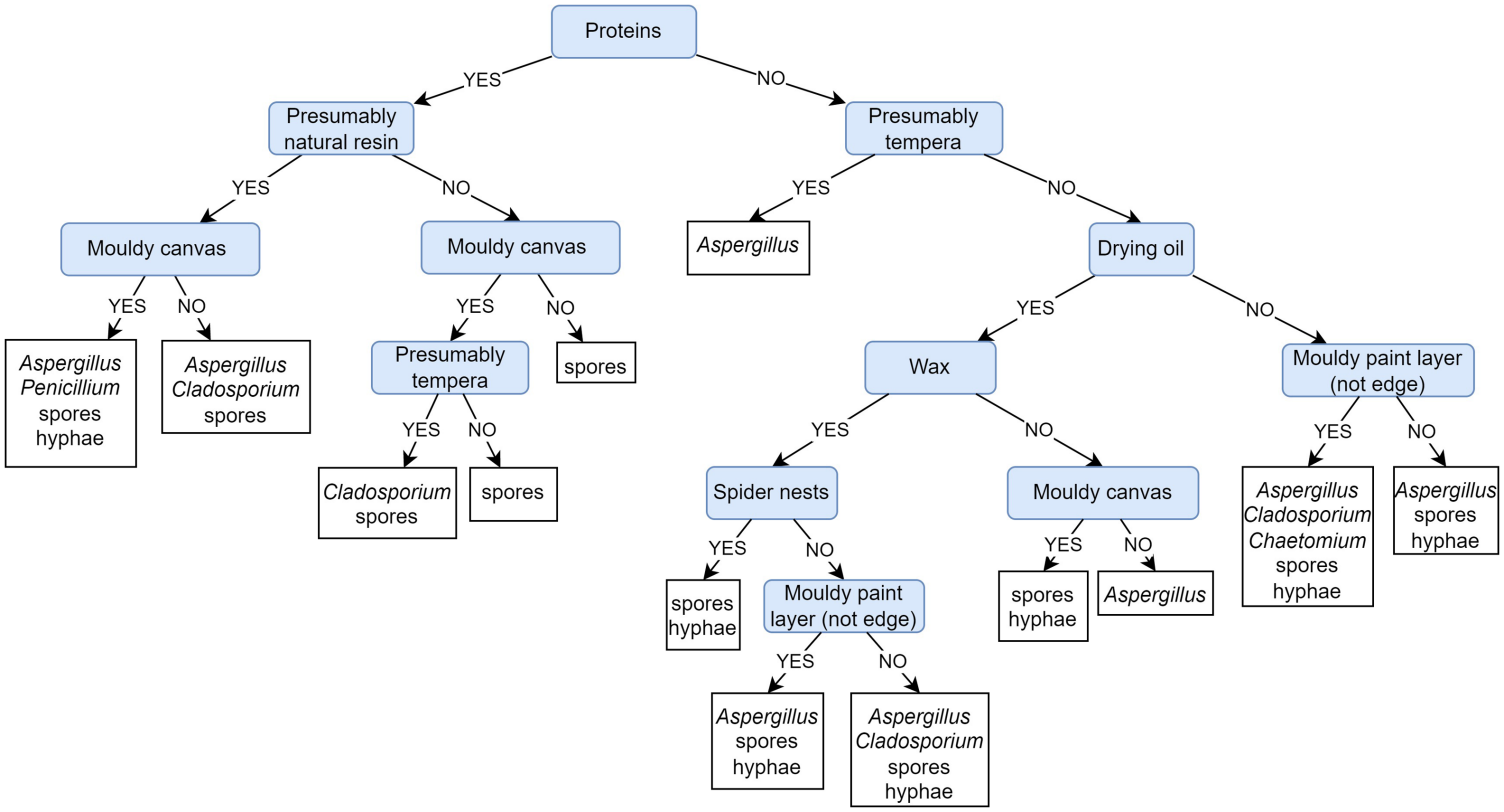
Predictive clustering tree (PCT) for multi-label classification (MLC), predicting the presence of on-site detected fungi based on the sampled paintings of known/unknown material composition. The decision process starts by separating paintings containing proteins. It continues with the presence of natural resins on paintings containing proteins and then diverges on the canvas sampling site. The painting technique is used for decision-making but is less important.

Figure 9 depicts a PCT model that predicts the presence of on-site recorded fungi based on the painting’s materials and sampling sites. Evaluation of the presented PCT and the default model shows that the weighted AUPRC of the presented PCT (0.94) is substantially improved over the weighted AUPRC of the default model (0.45). The PCT model and feature ranking expose the presence of proteins as the most important feature for the fungal colonization of paintings (Supplementary Table S5). When looking into the fungal community obtained on paintings containing proteins (left section of the PCT depicted in Figure 9), the presence of natural resins is further decisive. In addition to accumulated spores, the growth of *Aspergillus*, *Penicillium*, *Cladosporium,* and other fungi (unidentified hyphae) is predicted on such paintings: *Aspergillus* regardless of the side (back or front), *Penicillium* on the canvas, and *Cladosporium* on the painted side only. In the absence of natural resin, the latter can also appear on the canvas of tempera paintings. In the case of protein absence (right section of the PCT depicted in Figure 9), tempera paintings are the next decision step, which is likely to be overgrown by *Aspergillus* species. Only further on, the decision is based on the drying oil paintings, on which mycelium is predicted to grow regardless of the painting’s side and the presence/absence of wax and cobwebs. The genus *Chaetomium* is associated with moldy paintings in the absence of proteins in the canvas, but is not associated specifically with tempera or drying oil.

#### 3.7.2. Painting material composition and sampling sites in relation to isolated fungi

The PCT model that predicts the presence of isolated fungi based on material and sampling sites is presented in Supplementary Figure S1. The evaluation of the presented PCT and the default model shows that the weighted AUPRC of the presented PCT (0.47) is moderately improved over the weighted AUPRC of the default model (0.30). In contrast to the model obtained from the data on *in situ* detected fungi (Figure 9) in relation to painting materials, where the presence of proteins was exposed as the most important feature, paintings painted with (presumably) drying oil were the most important. From the model, it can be assumed that *Aspergillus* species can be isolated from paintings regardless of the paintings’ material since they appear in various leaf nodes throughout the tree. They can be associated with (presumably) drying oil and (presumably) tempera paintings; some species are predicted when the painting is protected with varnish and even impregnated with wax, and others without varnish cover. For example, the most frequently encountered *Aspergillus* species on paintings, *A. vitricola*, can appear on (presumably) drying oil as well as on other, not drying oil or tempera paintings, if covered with varnish and impregnated with wax; it can also appear on other paintings, such as tempera with wax, and proteins in the ground. *A. destruens* can be expected in culture from the paintings other than the ones assigned as presumably tempera and drying oil, not only in the presence of natural resins, wax, and proteins in canvas ground but also in the absence of greasy tempera and proteins in canvas ground and wax. *Cladosporium* and *Alternaria* can infest drying oil and tempera paintings, the latter if impregnated with varnish and wax; they are not associated with paintings impregnated with natural resins and proteins. The genus *Wallemia* occurs on greasy tempera paintings and on paintings other than (presumably) drying oil or tempera, on the back side, in the absence of proteins but impregnated with wax, which indicates its occurrence on animal fats (egg yolk and beeswax). Interestingly, it was not shown to be associated with proteins.

The performed feature ranking on the same dataset suggests that paintings painted with drying oil binders were the most colonized by fungi. Feature ranking also exposes proteins, presumably tempera, moldiness (back), and paint layer moldiness as the most important features (Supplementary Table S6).

#### 3.7.3. Painting damage in relation to microscopically detected fungi

**Figure 10:**
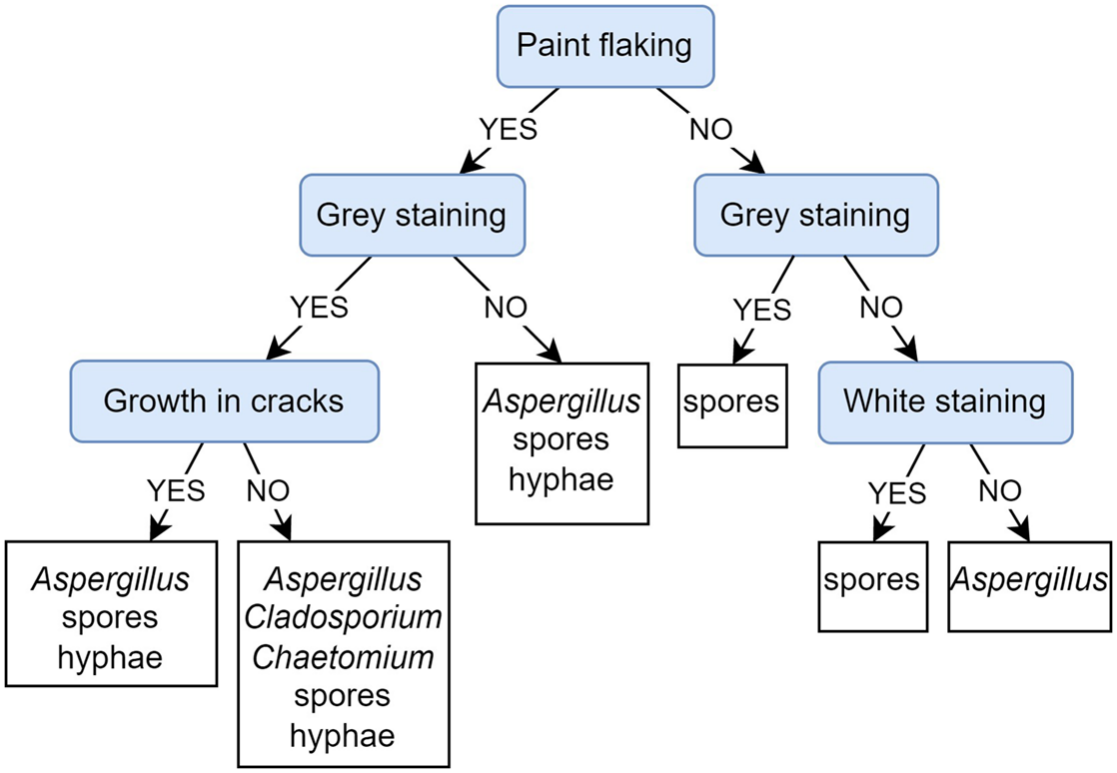
Predictive clustering tree (PCT) for multi-label classification (MLC), predicting the presence of fungal structures typical for certain genera or unidentified mycelia and spores, detected on the sampled paintings by microscopy. The decision process starts by separating paintings according to the presence/absence of paint flaking and then continues to observe staining and later cracking. The presence of mycelium is observed only in the left part of the PCT, while *Aspergillus* is also present in cases where flaking is not observed.

Figure 10 depicts a PCT model that predicts the presence of microscopically detected fungi based on observed damage to paintings. The evaluation of the presented PCT and the default model shows that the weighted AUPRC of the presented PCT (0.78) is substantially improved over the weighted AUPRC of the default model (0.45). The PCT highlights paint flaking as the first (most important) decisive factor to affect the fungal colonization of paintings. When looking into the fungal community at the flaking sites of paintings (left section of the PCT depicted in Figure 10), the mycelium as well as recognizable *Aspergillus* structures were observed in all cases regardless of the presence of gray stains; deeper in the tree, it is shown that the presence of mycelium can be associated with or without paint cracking. In cases where paint flaking was not observed (right section of the PCT, Figure 10), staining was associated with the presence of spores only.

**Figure 11:**
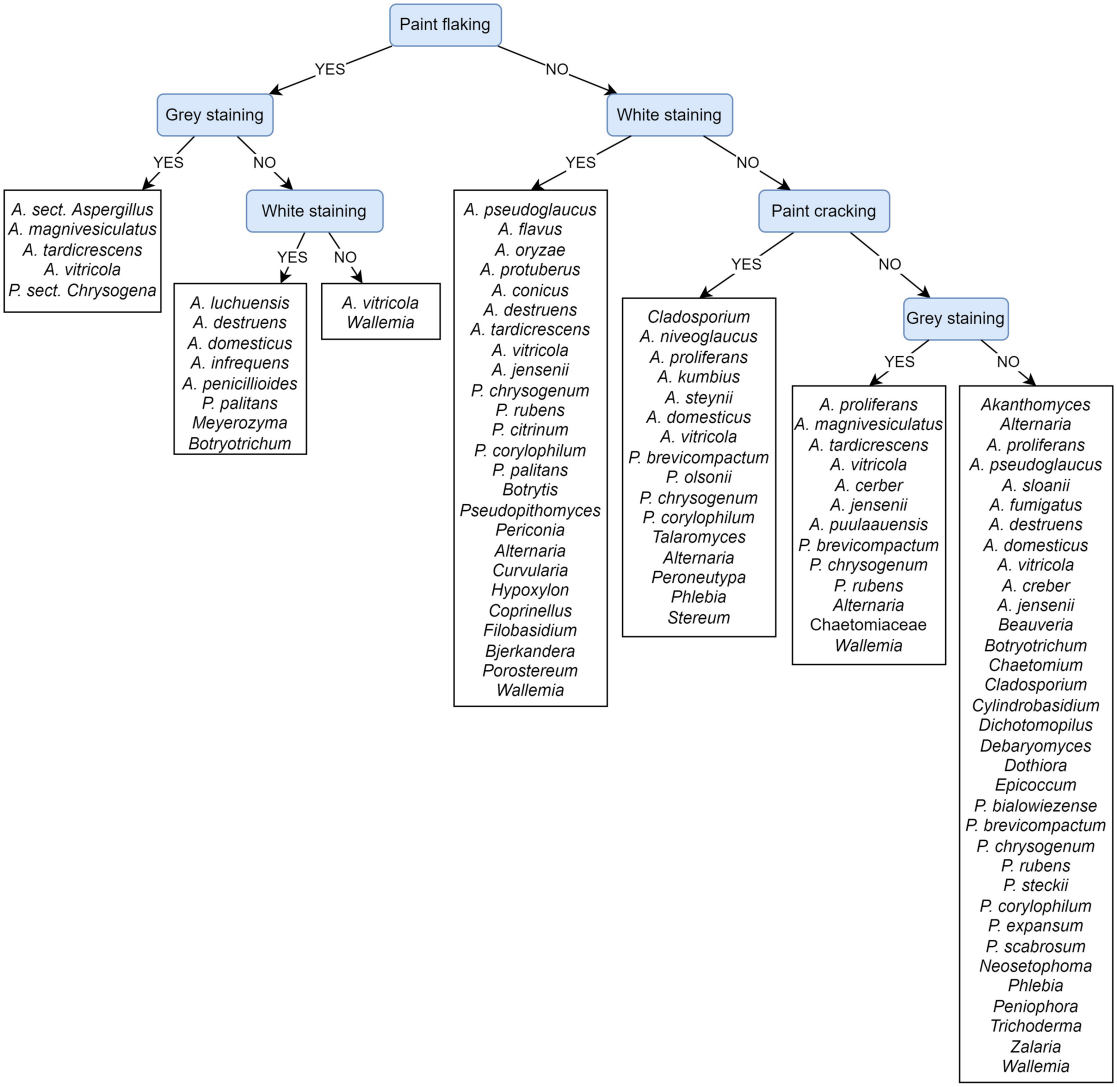
Predictive clustering tree (PCT) for hierarchical multi-label classification (HMLC), predicting the cultivation of fungal taxa from damaged areas of paintings. The decision process starts by separating paintings according to the presence/absence of paint flaking and then continues to observe staining and later cracking. Most of the prediction paths seem to predict a solid fungal presence.

Feature ranking exposes paint flaking and gray and white staining as the most important attributes. This result is in agreement with the PCT model presented on cultivated fungi from damaged areas (Figure 11), as well as with the corresponding feature ranking (Supplementary Table S7).

#### 3.7.4. Painting damage in relation to cultivated fungi

Figure 11 depicts a PCT model that predicts the cultivation of fungi from observed damage to paintings. The evaluation of the presented PCT and the default model shows that the weighted AUPRC of the presented PCT (0.37) is substantially improved over the weighted AUPRC of the default model (0.25). The prediction process starts by separating samples based on paint flaking and continues with the presence of stains. Paint flaking may or may not be associated with gray/white stains. *Aspergillus* species *A. magnivesiculatus*, *A. tardicrescens*, *A. vitricola*, and *P. chrysogenum* can be isolated from such areas, which also appear gray, while other *Aspergillus* species (*A. luchuensis*, *A. destruens*, *A. domesticus*, *A. infrequens*, and *A. penicillioides*), *P. palitans*, and some other species are associated with white stains. *Wallemia* and *A. vitricola* can also form other stains on paint flaking areas. Numerous species cause superficial white stains. Paint cracking appears only at the level of leaf no. 3 and is also associated with numerous *Aspergillus*, *Penicillium* species, and species of other genera (*Alternaria*, *Talaromyces*, and basidiomycetes genera *Peroneutypa*, *Phlebia,* and *Stereum*). Fungi of the genera *Akanthomyces*, some *Aspergillus* (*A. fumigatus* and *A. sloanii*), *Beauveria*, *Chaetomium*, *Dichotomopilus, Dothiora*, *Epicoccum*, some *Penicillium* (*P. bialowiezense*, *P. steckii*, *P. expansum*, and *P. scabrosum*), *Peniophora,* and *Zalaria* were found exclusively on paintings without the visible damages described in the tree (e.g., paint flaking and cracking) and were not associated with white and/or gray stains. Fungi of the genera *Aspergillus* (*A. creber*, *A. destruens*, *A. domesticus*, *A. jensenii*, *A. proliferans*, *A. pseudoglaucus*, and *A. vitricola*), *Alternaria*, *Botryotrichum*, *Cladosporium*, *Penicillium* (*P. brevicompactum*, *P. chrysogenum*, *P. rubens*, and *P. corylophilum*), and *Phlebia*, *Wallemia*, were found on the paintings in the presence and absence of observed physical damage (paint flaking and cracking) and staining.

Different from the PCT model, feature ranking exposes paint cracking as the most important attribute, followed by paint flaking and white and/or gray staining (Supplementary Table S8).

#### 3.7.5. Potential relation of *Aspergillus* species with materials and observed damage on paintings

Because *Aspergillus* is the most abundant genus among the paintings, we also examined its possible relationship to the materials and its impact on the observed damage to the paintings. Supplementary Figure S2 shows a PCT model that predicts the presence of *Aspergillus* species based on the observed damage. Evaluation of the presented PCT and the default model shows that the weighted AUPRC of the presented PCT (0.87) is substantially improved over the weighted AUPRC of the default model (0.50). The most important features are gray stains, while the presence of greasy tempera (left part of the tree presented in Supplementary Figure S2) and proteins (right part of the decision tree) are secondary features. Most of the leaf nodes do not contain *Aspergillus* species, but a few will still be revealed in the third leaf, such as *A. reticulatus* being associated with gray stains and not with cracking, or other species deeper in the tree (*A. destruens* and *A. jensenii* associated with wax; *A. pseudoglaucus* with proteins). These are not predicted to cause important physical damage, such as cracking, peeling, tearing, or other aesthetic damage. Different from the PCT model, feature ranking exposes presumably greasy tempera as the most important attribute, followed by the presence of proteins (Supplementary Table S9).

## 4. Discussion

Easel paintings are rich in organic materials and therefore support the growth of heterotrophic organisms such as saprotrophic bacteria and fungi. Microbiological studies generally focus either on bacteria (e.g., Pavić et al., 2015) or fungi (e.g., Sabatini et al., 2018), while some studies address both microbial groups (e.g., Capodicasa et al., 2010; Caselli et al., 2018; Poyatos-Jiménez et al., 2021). Regardless of the nutritional status of the substrate, fungi in particular play an important role in the biodegradation of historic paintings stored indoors because they can grow at a lower RH compared to bacteria and potentially cause damage (Dannemiller et al., 2017). Therefore, we focused on xerotolerant and xerophilic fungi.

We describe fungal communities from 24 easel paintings sampled in the past 10 years. Different sampling tools, including Copan swabs and RODAC plate prints, were used for some paintings and sterile cotton swabs for all. Fungal structures were microscopically detected *in situ* whenever possible. Selected isolation media targeted especially xerotolerant and xerophilic species. *In situ* detection of fungal mycelium on paintings implies that fungi actively colonize and degrade paintings.

### 4.1. Several criteria have to be considered to address problematic contaminants in paintings

Due to the omnipresence of fungal propagules in the air (Awad et al., 2020) and deposited dust (Amend et al., 2010; Visagie et al., 2014), it is challenging to identify problematic species growing on painting by applying culture-dependent or culture-independent techniques. Both can lead to an overestimation of species producing spores in a studied niche (López-Miras et al., 2013b). For this reason, we also applied other techniques to confirm active contaminants. We applied the following two: microscopical *in situ* detection of mycelium on tape prints and cultivation on culture media representing substrates with low a_w_ mimicking conditions of low RH indoors.

In attributing the active presence of fungi to paintings, it is difficult to follow a single concept since paintings are sampled at one point in time. However, certain contaminants might have been present already on paintings for a long time, or primary colonizers (still present, e.g., as resting spores) might have been replaced with secondary contaminants. Chemical changes in microhabitats may also lead to altered microbial community structures (Ciferri, 1999). All this is hardly possible to deduct in single-time sampling. Several aspects can be tackled: (i) how to recognize fungal infestation, (ii) how to distinguish them from dust- and air-deposited structures, and (iii) how to find active contaminants and distinguish them from past inactive contaminants. Although advanced software analysis tools allow human-independent recognition of areas of paintings decayed by microorganisms, e.g., *MicroorganismPattern* software (Calderón-Mesén et al., 2023), decayed areas were identified following “naked-eye” inspections or with the help of an USB microscope. To confirm the infection, we used optical microscopy of tape prints as a non-destructive method of sampling in most of the cases in our study. This method allowed us to identify the spatial distribution and morphology of the colonizing microorganisms (Urzi and De Leo, 2001). Tape print also allowed the detection of mycelium, indicating the active presence of fungi on the painting surface at one point in time. Coupling tape print microscopy with fluorescent dyes allows discrimination between active and dead species (Okpalanozie et al., 2016). In our study, the active contaminants were confirmed by cultivation, where we did not consider species growing only in single colonies to avoid dust/air contaminants. The tape print technique also allowed the identification of fungi to the genus level according to the observed structures. *Aspergillus*, detected in 82% of sampled paintings, was the most often detected genus on paintings. It was detected in 83% of cases by cultivation. Other studies on paintings reported *Aspergillus*, *Penicillium*, *Cladosporium*, and *Alternaria* as the most frequently encountered genera based on morphology (e.g., Caselli et al., 2018). Our findings agree with those, especially concerning *Aspergillus* and *Cladosporium*-like infestations. *Penicillium* and *Alternaria* species were relatively rarely recognized on tape prints in our study, indicating the possibility that they represent secondary colonizers.

### 4.2. Culture media adapted to environmental RH are necessary to detect indoor biodeterioration agents in cultures, further enabling identification and *in vitro* testing

Cultivation remains an important component for analyzing possible biodeterioration agents as it allows applying standardized morphological characterizations and sequencing phylogenetic marker genes for fine-tuned identifications at the species and infraspecific level. It also enables the use of existing knowledge since the species name can provide a link to published information (Houbraken et al., 2020). In addition, pure cultures enable *in vitro* testing of degradation abilities and the testing of antimicrobial treatments.

Prior to cultivation, the sample has to be taken from the painting. Sampling of cultural heritage objects in general is supposed to be non-invasive. Therefore, different approaches have been adopted so far. Originally and still often used cotton swabs (Vieto et al., 2022) were in some studies replaced by rayon swabs (Caselli et al., 2018) or nitrocellulose membrane prints (Okpalanozie et al., 2016; Văcar et al., 2022). Some researchers used adapted vacuum systems to collect particles (de Carvalho et al., 2019). In our study, we used standard cotton swabs in all paintings. In two paintings, additional nylon brush swabs (Copan ESwab^™^ collection and transport system) were used. Cotton swabs have some restrictions, e.g., in cases of severe infestations, they may result in the application of overloaded inoculum not allowing separating colonies or when spores of certain fungi might become trapped in cotton fibers and are not released onto culture media or into liquids. Although the application of liquid is better for inoculum dispersal, it might affect spore survival due to unadjusted osmolarity to the environmental RH values. Therefore, sampling with cotton swabs still provides a backup for retrieving fungal contaminants active at extremely low RH situations, while the Copan system provides better spreading of inoculum, which could be particularly important in quantitative studies The RODAC plates were only used in a few cases as they left a wet stain on the surface.

When applying samples to culture media, researchers have so far followed the methods of medical mycology and treated paintings as “patients,” most often using culture media such as Sabouraud Glucose Agar (SGA; Sabatini et al., 2018; Calderón-Mesén et al., 2023) or fungal Trypticase Soy Agar (TSA; López-Miras et al., 2013a) for bacterial inventories. Recently, more advanced approaches have used CMC cellulose as a carbon enrichment in isolation media. Its use was based on the assumption that the canvas support is the most important substrate for fungi infesting paintings (Vieto et al., 2022). Media with a low a_w_ target fungi in low RH environments such as storage facilities including musea, archives, and libraries (Micheluz et al., 2015; Skóra et al., 2015; Bastholm et al., 2022), cultural heritage sites with salt deposits (Martinelli et al., 2017), and indoor environments (Nevalainen et al., 2015). Therefore, paintings stored indoors, either in uncontrolled and therefore changing environmental conditions, e.g., in churches, castles, other old buildings, or in modern storage rooms following expert recommendations (16°C–18°C, 40%–65%; Kavkler et al., 2015), should be examined for the presence of low-water activity-thriving, xerophilic microorganisms. The necessity of this approach is well illustrated in this study by the concurrent use of culture media, such as general isolation and enumeration medium DRBC, which in many cases harbored only a few colonies, in contrast to DG18, MY10-12, and MY50G, with almost confluent growth when applying the same sample (Figure 5). In relation to temperature, RH determines the dew point, enabling the formation of water droplets that allow the initiation of the biodeterioration process in the case of microbial load (Berovič, 2003), which also continues in lower water availability conditions. This is confirmed by the results of this study, in which so far not yet reported xerophilic species of *Aspergillus* section *Restricti* and *Wallemia* species were among the most frequently seen isolates. The majority of these species were not yet identified from paintings. *A. vitricola* was reported from museum outbreaks and wooden organs (Sterflinger et al., 2018), book libraries (Micheluz et al., 2015), and a depository in Denmark (Bastholm et al., 2022). *Aspergillus penicillioides* was reported frequently from archives (Borrego et al., 2017; Pinheiro et al., 2019) and from museum items (Liu et al., 2018), but not in earlier studies (Nittérus, 2000). Xerotolerant contaminating taxa were detected on high a_w_ media. These are species of *Aspergillus* section *Aspergillus* (formerly classified in *Eurotium*) (Hubka et al., 2013), species of *Aspergillus* section *Versicolor* (Jurjević et al., 2012), *Cladosporium*, *Alternaria* (Caselli et al., 2018), and also other genera.

### 4.3. Biodeterioration potential of xerophilic species

The genera *Aspergillus* and *Penicillium* include many species that have attracted attention due to their exceptional biodeterioration potential, as many have been shown to be good producers of pigments, acids, and various extracellular enzymes (Savković et al., 2019). The threat to cultural heritage posed by *Aspergillus* was recently investigated (Romero et al., 2021); among the 52 species considered, only three xerophilic species were documented: (i) *A. penicillioides*, frequently detected on paper collections but also on canvas, wood, leather, historic stained glass, and on a mural and (ii) *A. restrictus*, known from historic stained glass and from the Hallstatt wooden staircase (Martinelli et al., 2017). Along with these two species, other species may be recognized, which were later described as new due to the broader species concept adopted in the past; (iii) *A. halophilicus*, known from books stored on shelves, paintings on paper, wood, leather, and gelatin silver photographs (Romero et al., 2021). Of all these three species, only *A. penicillioides* was also among the isolates in our study. However, while our two most common species, *A. vitricola* and *A. destruens*, are not mentioned in this review, *A. vitricola* was detected on a historic pipe organ in a church but listed as a potentially new *Aspergillus* sp. by Sterflinger et al. (2018). Both species are known from indoor air; *A. destruens* also from the surface of cheese, indicating a preference for proteinaceous and/or lipidous substrates, and from maize seeds (Sklenář et al., 2017). *Aspergillus vitricola*, originally described from a binocular lens, is most commonly recovered from house dust and has already been found on archival material (Sklenář et al., 2017). Isolates of *A. tardicrescens* are known from museum pieces of various materials (rubber, glass, and others), from air and house dust, and from *A. magnivesiculatus* from house dust and dry corn (Sklenář et al., 2017). Morphologically and physiologically, these species are hardly distinguishable; some species of the *Restricti* series even resemble the distantly related *A. fumigatus* because of the columnar conidial heads. Most species of the section *Restricti*, with the exception of *A. penicillioides* and some strains of *A. destruens*, cannot grow on media without additional solutes (a_w_ approximately 0.99) (Sklenář et al., 2017). A very high and unique chemical diversity of secondary metabolites was observed in the section *Restricti*, e.g., all species produced asperglaucide, asperphenamate, or both (Sklenář et al., 2017) and simple phenylalanine-derived compounds with anticancer activity (Bladt et al., 2013). Both species have differing beta-tubulin sequences, the commonly used molecular marker for identification. Within *A. vitricola*, there is up to 10% variability between sequences of different strains (Sklenář et al., 2017), which is why some researchers consider certain isolates undescribed species when comparing them to the type strain (Sterflinger et al., 2018). The *A. destruens* isolate from a painting of this study is known for its halophilic nature and ability to degrade cyclic carbohydrates under saline conditions (González-Abradelo et al., 2019). It also has the ability to degrade paints (Kosel et al., 2021). Of concern is its potential to create more favorable conditions for less xerophilic fungal species to grow, metabolize substrates, and produce potentially dangerous mycotoxins. In this regard, we can explain the presence of numerous xerotolerant isolates reported from our and other studies. *Aspergillus penicillioides* has been reported to be metabolically active at the lowest levels of water activity ever studied (e.g., a_w_ 0.585), which corresponds to a RH of 58.5% (Stevenson et al., 2017). It is closely related to an isolate we obtained from a painting; however, we have not yet tested the growth of our isolate on media with similarly low-water activities. Metabolic water at such low-water activities increases the water activity of the substrate and allows other species to grow (Sklenář et al., 2017). The changing water activities in the substratum lead to succession events that can be unique, depending on material and environmental conditions.

All xerophilic *Aspergillus* species identified in our study had halotolerance and xerotolerance traits previously reported (Sklenář et al., 2017). Furthermore, Savković et al. (2019) previously tested the *in vitro* biodeterioration potential of several *Aspergillus* species, including *A. domesticus*, *A. penicillioides*, *A. pseudoglaucus*, and *A. ruber*, which were also isolated from paintings in our study. Many of the tested fungi demonstrated significant deteriorative ability, indicating their potential to cause structural and aesthetic alterations in the paintings from which they were isolated.

Species of *Wallemia*, known from saline environments (Jančič et al., 2016), proved to be an important contaminant of paintings. Due to their slow growth, they can be easily overlooked; therefore, low-water activity culture media and the presence of salt are also key factors. The prediction model in Supplementary Figure S1 connects the presence of *Wallemia* with wax or animal fat but not to protein. It is surprising that among the eight described species, only two are associated with paintings: *Wallemia* aff*. muriae*, which still requires in-depth study of taxonomic markers to be correctly identified, and *W. canadensis*, while several isolates are listed as *Wallemia* sp. *Wallemia* aff*. muriae*, isolated from painting RCS 20, grew from a swabbed front area of a waxed drying oil painting.

### 4.4. Existing data on the occurrence of xerophilic fungi obtained by culture-independent methods

Culture-dependent techniques and confirmed *in situ* detections identified xerophilic *A. destruens*, *A. vitricola*, and *Wallemia* aff. *muriae* as active fungal contaminants on damaged paintings. *Aspergillus tardicrescens* and *A. magnivesiculatus*, here newly detected on paintings, contributed to mixed infections together with xerotolerant *A. proliferans* (Kavkler et al., 2015; Borrego and Molina, 2019), *A. jensenii* (Kavkler et al., 2015), and *A. versicolor* (Sterflinger, 2010), and species of *Cladosporium* (Calderón-Mesén et al., 2023).

The separation of active contaminant species on paintings from dust and airborne flora is also a challenge for culture-independent methods. Illumina short amplicon sequencing targeting the fungal Internal Transcribed Spacer 2 region (ITS2) (de Carvalho et al., 2019) and whole-genome amplifications (WGA) with the Nanopore sequencing technology (Piñar et al., 2020) provided huge amounts of valuable data. In both studies, *Ascomycota* was the dominant phylum, with the genus *Aspergillus* detected in all samples, as in our study. Due to short reads and restricted taxonomic resolution of the ITS region, Illumina short amplicon sequencing has the disadvantage of not being able to make species-level identifications, specifically for the most frequently detected genera on paintings, such as *Aspergillus*, *Cladosporium*, and *Penicillium*. The WGA protocol based on nanopore sequencing allowed the identification of microorganisms; however, identified *Aspergillus fumigatus* and *A. glaucus* (Piñar et al., 2020) have hardly been detected in our study, although we studied the same kind of deterioration phenomena. Since culture-independent methods still have some drawbacks (e.g., DNA isolation from spores), they need to be coupled with cultivation techniques (Liu et al., 2018). Another analysis of settled dust in a library by the LC-MS/MS system detected a wide range of toxic or bioactive fungal metabolites. Particularly high was the contribution of asperglaucide (Micheluz et al., 2016), which is indicative of xerophilic *Aspergillus* species from section *Restricti* (Sklenář et al., 2017). Accordingly, the study by Micheluz et al. (2016) is congruent with the results we obtained. *Aspergillus* species from section *Restricti* obviously play an important role as contaminants in easel paintings.

### 4.5. Can machine learning methods from obtained data give us new insights about important contaminants?

Just 24 sampled paintings with different supports, glues, adhesives, paints, and storage/exhibition conditions, along with information about detected and isolated fungi, represent an almost unmanageable amount of data. Often, certain information was missing, which limited the use of classical statistical methods. Machine learning methods can deal with such datasets and have already been used in ecological studies. They pinpointed some relationships that classical methods would not have unraveled easily (Jančič et al., 2016; Kavkler et al., 2022; Novak Babič et al., 2022). To obtain more comprehensive and reliable predictive models, such analyses require larger, consistent, and feasible datasets, which are essential for ensuring the accuracy and robustness of the analyses. Nonetheless, the methods employed provided valuable insights into the potential connections between fungi, materials, and resulting damage.

We analyzed several datasets to obtain predictive models for fungal occurrence on paintings. With respect to materials associated with painting techniques and observed damage, and using data on the presence and isolation of fungi from paintings, the models predict that *Aspergillus* spores and mycelia can be expected on all surfaces of paintings. By means of contrast, species of *Cladosporium* are more likely to occur on the backs of paintings. In general, proteins are the main factor in the fungal colonization of paintings. They may be of animal or plant origin and are added to canvas as glues and sizing agents (Ciferri, 1999). *Aspergillus* and *Cladosporium* species may also be present on paintings treated with natural resins, which are considered by some authors to be more difficult to degrade (Poyatos et al., 2018). Waxed oil paintings that do not contain proteins are also likely to contain *Aspergillus*, *Cladosporium*, and the mycelia of other fungi.

According to the analyses of the isolated fungal community in relation to the material composition of the paintings and in contrast to the fungi detected *in situ*, drying oil paintings were at the top of the decision tree, while proteins are less important for supporting fungal growth on paintings. In terms of damage and stains on paintings, according to the PCT analysis, paint flaking is the most decisive factor for fungal infestations. However, according to the feature ranking, paint cracking is the most decisive. *Aspergillus* structures and other unidentified mycelia may be observed *in situ* at these damaged areas, may or may not be associated with visible stains, and may even grow in cracks, as seen in paintings RCS 25 and RCS 26. *Cladosporium* and *Chaetomium* structures were also observed on painting surfaces with peeling paint, but unlike *Aspergillus*, they are not expected to grow in cracks. The PCT analysis, based on isolated and identified fungi from damaged areas, predicts the isolation of numerous species of the genera *Aspergillus* and *Penicillium*, as well as other fungi. Some species are expected to be present on all damages listed in the leaves of the tree shown in Figure 11. *Aspergillus vitricola*, *P. chrysogenum*, and others may occur on more specific sites, *A. magnivesiculatus* on paint flaking sites, *A. domesticus* on paint flaking and paint cracking sites, and *A. tardicrescens* and *Cladosporium* spp. on paint cracking sites. However, it is likely that all of these taxa are also present on-sites without damage, indicating either their dispersal by air currents or by insects, as confirmed by the presence of cobwebs and insect-related taxa such as *Akanthomyces* and *Beauveria*. On the other hand, the painting may also represent a growth surface whose component is not used for fungal nutrition, where fungi may feed simply on environmental debris (Ciferri, 1999).

*Aspergillus* is overall the most abundant genus and was detected both by *in situ* microscopy and by cultivation in damaged areas. We also isolated *Aspergillus* from visibly undamaged areas of the paintings. According to our model shown in Supplementary Figure S2, *Aspergillus* is not predicted to cause physical damage, which is in conflict with the model shown in Figure 11, where certain *Aspergillus* species are associated with paint flaking, cracking, and staining.

Consistent with the data observed during microscopic examination and cultivation, even machine learning methods do not identify any specific taxa associated with a particular material or type of damage. Similarly, the presence of the genus *Aspergillus* is predicted for almost all situations, while specific species are rarely predicted to be associated with a particular problem, such as *A. reticulatus* producing gray spots on presumably greasy tempera paintings. Further down the decision trees, we see some associations of *Wallemia* spp., *Aspergillus destruens,* and *A. vitricola* with wax, a material traditionally used in restoration practice.

If these data were supplemented with data from other examined paintings, the predictive value of such models would increase.

### 4.6. Recommendations for further studies

The biodeteriorative potential of newly recorded species on paintings needs to be investigated on mock-up samples, and methods to mitigate their growth need to be explored. The susceptibility of restoration materials needs to be investigated for xerophilic fungi. New environmental measures to reduce the attack of xerophilic fungi on paintings and other historic materials need to be considered, as has been emphasized for libraries and paper heritage storage (Bastholm et al., 2022). Special attention should also be paid in further studies of deteriorated paintings to the selection of culture media, such as DG18, MY50G, and MY10-12, to obtain xerophilic fungi and to develop new culture media targeting additional saprotrophic groups acting at low a_w_, such as oil-degrading fungi that can grow under low RH conditions. A database on damaged paintings, collecting all available data on damages, materials, and fungi, should be built for the application of machine learning methods to provide more accurate predictive models.

## 5. Conclusion

This study reports on 24 deteriorated historical paintings that were examined for the presence of fungi using microscopy and culture-dependent techniques. Using selected media for xerotolerant/xerophilic species, 465 fungal strains belonging to 37 genera and 98 species were found. Xerophilic species of the genus *Aspergillus*, including some species newly discovered on paintings in this study, dominated over xerotolerant mycobiota, regardless of the side of the painting. Xerophilic *Wallemia* species and xerotolerant members of *Aspergillus*, *Cladosporium*, and *Penicillium* accompanied the presumed primary colonizing xerophilic species on painting surfaces. Machine learning approaches imply that *Aspergillus* species are expected to be isolated from all materials and damaged areas; however, some may require special culture media. Proteins in paintings are generally among the most important factors for mold growth, while wax may allow the growth of certain taxa, such as *Wallemia*. Cracks in the paint layer create hotspots for mycelial development, which can accelerate the process of biodegradation.

## Data availability statement

The datasets presented in this study can be found in online repositories. The names of the repository/repositories and accession number(s) can be found in the article/Supplementary material.

## Author contributions

PZ: Conceptualization, Data curation, Funding acquisition, Investigation, Methodology, Supervision, Validation, Writing – original draft. DGH: Writing – review & editing, Investigation. CG: Investigation, Writing – review & editing, Methodology, Visualization. MB: Investigation, Methodology, Visualization, Software, Writing – original draft. SD: Methodology, Writing – review & editing. MM: Writing – review & editing, Investigation. MNB: Writing – review & editing, Formal analysis. JČZ: Writing – review & editing, Data curation. AK: Writing – review & editing, Investigation. NG-C: Writing – review & editing, Funding acquisition. KK: Writing – review & editing, Conceptualization, Investigation, Methodology.

## Footnotes

^1^ http://www.ex-genebnk.com/
