# Supplementary material for: Xerophilic fungi contaminating historically valuable easel paintings from Slovenia
Source: Front Microbiol. 2023 Nov 2;14:1258670. doi: 10.3389/fmicb.2023.1258670 (PMC10653331; doi:10.3389/fmicb.2023.1258670)

Supplementary Material

Xerophilic fungi contaminating historically valuable easel paintings from Slovenia

Polona Zalar^1,*^, Daša Graf Hriberšek^1^, Cene Gostinčar^1^, Martin Breskvar^2^, Sašo Džeroski^2,3^, Mojca Matul^1^, Monika Novak Babič^1^, Jerneja Čremožnik Zupančič^1^, Amela Kujović^1^, Nina Gunde - Cimerman^1^, Katja Kavkler^4^

*** Correspondence:** Polona Zalar: [polona.zalar@bf.uni-lj.si](mailto:polona.zalar@bf.uni-lj.si)

# Supplementary tables

## Supplementary Table 1: List of paintings examined with available information on provenance and location, presumed or confirmed materials and painting techniques, and dates of sampling.

| **Painting** | **dating; author: »painting title«** | **Size;**  **institution, location; storage** | **Sampling dates** | **Organic painting constituents** | **Technique** |
| --- | --- | --- | --- | --- | --- |
| RCS 15 | presumably 1726; unknown author:  »3^rd^ scene from the life of St. Francis« | 220×210 cm;  church, Ljubljana; ZVKDS | 27.02.2012,  18.09.2014,  5.03.2015  27.08.2015 | flax canvas, drying oil, wax | oil |
| RCS 16 | presumably 1726; unknown author:  »4^th^ scene from the life of St. Francis; | 220×220 cm; church, Ljubljana; ZVKDS | 27.02.2012 | flax canvas, proteins (canvas isolation), drying oil, maybe tempera, natural resin (varnish), proteins (ground layer) | unidentified (oil or greasy tempera) |
| RCS 17 | unknown dating and author:  »St. Francis Xavier« | 233×177 cm; church,  Novo mesto; ZVKDS | 27.02.2012 | hemp canvas, proteins (ground layer), oil (paint), natural resin (varnish) | oil |
| RCS 18 | unknown dating and author: »Crucifixion« | 202,5×108,5 cm; Church,  Novo mesto; ZVKDS | 27.02.2012 | hemp canvas, drying oil (ground and paint layers), wax | oil |
| RCS 19 | 1931; Tone Kralj: »Stations of the Cross, 1^st^ station« | 106×70 cm; Church, Mengore, ZVKDS | 27.02.2012 | flax canvas, drying oil, wax, cellulose derivates (restoration) | oil |
| RCS 20 | unknown dating and author:  »Mary's engagement« | 238,5×149,5 cm; Church, Idrija; National gallery, ZVKDS | 18.09.2014  5.03.2015 | unidentified canvas, wax, drying oil | oil |
| RCS 21 | 18^th^ century; unknown author: »John of Nepomuk« | 201×133 cm; Monastery, Ajdovščina; National gallery, ZVKDS | 5.03.2015 | flax canvas, wax, drying oil, maybe greasy tempera | unidentified (oil or greasy tempera) |
| RCS 22 | 17^th^ century; unknown author: »Madonna of the Rosary (Rožnovenska Mati Božja«) | 79,5×129 cm; Monastery, Ajdovščina; National gallery, ZVKDS | 27.02.2012,  18.09.2014,  5.03.2015,  22.07.2020, 7.09.2020 | hemp canvas original, flax canvas later added, greasy tempera, maybe drying oil, starch | unidentified (oil or greasy tempera) |
| RCS 23 | unknown dating and author: »St. Barbara and St. Ahac with saints«  (Sv. Barbara in sv. Ahac s svetniki«) | 316,5×160,5 cm; Church, Idrija; National gallery, ZVKDS | 5.3.2015 | flax canvas, drying oil,  greasy tempera | oil and greasy tempera |
| RCS 24 | unknown dating; Matej Ingoli:  »Worship of St. Cross« »Čaščenje sv. Križa« | 327×160 cm; Church, Izola; National gallery, ZVKDS | 5.3.2015 | flax canvas, wax, drying oil, proteins | oil |
| RCS 25 | unknown dating and author: »Scene 5 (from the life of St. Francis) « | 216×211 cm; Church, Ljubljana; ZVKDS RC | 5.3.2015 | flax canvas, wax, maybe natural resin, drying oil, proteins | oil |
| RCS 26 | unknown dating and author: »Scene 2 (from the life of St. Francis) « | 216×211 cm; Church, Ljubljana; ZVKDS RC | 5.3.2015 | flax canvas, natural resin, drying oil | oil |
| RCS 36 (CB 8) | unknown dating and author: »St. Blaz as a bishop« | 70×50 cm; Church, Bled | 12.9.2016 | unknown canvas, oil or tempera | unidentified (oil or greasy tempera) |
| GBJ 1 | 1966; Polde Oblak: »Hostages« | 170×170 cm; Božidar Jakac Art Museum, Kostanjevica na Krki | 26.6.2015 | plywood, oil | oil |
| GBJ 2 | 1981; Jože Marinč: »Landscape« | 90×130 cm; Božidar Jakac Art Museum, Kostanjevica na Krki | 26.6.2015 | unknown canvas, oil | oil |
| GM 1 | 20^th^ Century; Fedja Žbona (unknown title) | 100×115 cm; Regional Museum Goriški muzej, Solkan | 1.10.2015 | flax canvas, acryl | acrylate |
| GM 2 | 20^th^ Century; Rafael Nemec: »Battle of the Giants« | 80×121,5 cm; Regional Museum Goriški muzej, Solkan | 1.10.2015 | veneer, oil | oil |
| AIO 1 | 1980/90; Aleksa Ivanc Olivieri: (unknown title) | 80x100 cm; private collection, Corsica, France; MG+MSUM Ljubljana | 28.10.2014 | unknown canvas, drying oil | tempera |
| AIO 2 | 1965; Aleksa Ivanc Olivieri: »The Birth of Light« | 78x94 cm; private collection, Corsica, France; MG+MSUM Ljubljana | 28.10.2014 | unknown canvas, drying oil | oil |
| AIO 3 | 1986; Aleksa Ivanc Olivieri: »The Golden Landscape« | cca. 210×150 cm; private collection, Corsica, France; MG+MSUM Ljubljana | 28.10.2014 | unknown canvas, drying oil | oil |
| ART-1 | 1516; Vittore Carpaccio: »Virgin and Child amidst a group of saints« | 415×252,5 cm; Church, Koper | 17.10.2019 | flax canvas, proteins (ground layer), drying oil (paint), natural resins | oil |
| ART-2 | 1537, Girolamo da Santacroce: »Mary with a Child and two Saints« | 180×137,5 cm; Church, Izola | 17.10.2019 | unknown canvas, drying oil (original paint and ground layer and retouching paint), proteins (restoration; original paint layers), natural resin (restoration); restored in 2005 | oil |
| ART-4 | 18^th^ Century; unknown author and title | cca. 200×90 cm; Church, Leskovec at Višnja gora | 13.10.2020 | unknown canvas, drying oil | oil |
| ART-5 | 1756; Anton Cebej: »St. Izidor«; painting restored in 1990 | cca. 300×100 cm; Church, Trška Gora | 13.10.2020 | unknown canvas, drying oil, restored in 2008 | oil |

Legend: (**CC**) Catholic Church, Institute for the Protection of Cultural Heritage of Slovenia, Restoration Centre (ZVKDS). (**MG+MSUM**) Modern galery, Metelkova Museum of Contemporary Art, Ljubljana

## Supplementary Table 2. Microscopically documented or/and isolated fungi from described sampling spots of paintings. The predominant isolated species on the paintings are in bold. Painting codes are explained in Suppl. Table 1.

| Painting code | Sampling site | fungi/fungal structures detected by microscopy | Isolated fungal species |
| --- | --- | --- | --- |
| RCS 15 | front, mouldy paint | black septate hyphae | ***A. destruens****, Wallemia* aff. *muriae* |
|  | back, mouldy glued canvas with cobweb | conidia of *Aspergillus* (5.2×3.7 µm, 2.5×1.7 µm), *Cladosporium* (6.5×2.7 µm); laterally budding hyphal fragments | *A. creber, A. fumigatus,* ***A. destruens****, A. jensenii, A. vitricola, P. corylophilum, P. rubens, D. hansenii* |
|  | front, non-mouldy **control** | *-* | *A. destruens, A. domesticus, A. vitricola, P. bialowiezense* |
| RCS 16 | back, mouldy green stain at the glued canvas | */* | *A. creber,* ***A. destruens****, A. sloanii, Chaetomium subaffine* |
|  | back, white mouldy stain | */* | *A. conicus, A. destruens* |
| RCS 17 | front, grey powdery paint | **/** | ***A. destruens****, A.conicus, A. puulaauensis* |
| RCS 18 | back, grey and black stains, thinned canvas | / | *Chaetomium cochliodes, Botryotrichum domesticum* |
| RCS 19 | wooden frame, white spots | **/** | ***A. jensenii****, Wallemia* aff. *muriae* |
|  | back, canvas | **/** | ***A. creber****, A. puulaauensis, Wallemia canadensis* |
| RCS 20 | front, grey and brown stains, cracked paint layer | conidia of *Aspergillus* (4.7×3.7 µm); unidentified conidia (3.8×3.2 µm); mycelium | ***Wallemia*** aff. ***muriae*** |
|  | back, white mycelium | **/** | ***A. vitricola****, Wallemia* aff. *muriae, Aspergillus* sp. |
| RCS 21 | front, cracked and flaking paint layer, grey stains in the bottom right corner; peeling paint layer | conidia of *Aspergillus* (4.0×3.5 µm); unidentified conidia (3.5×3.0 µm); mycelium | *A. glaucus, A. magnivesiculatus, A. reticulatus, A. tardicrescens, A. vitricola* |
|  | back, grey-white spots at the bottom | conidia of *Cladosporium* (7.8×3.5 µm); unidentified conidia (4.1×3.8 µm); mycelium | *A. creber, A. domesticus, A. magnivesiculatus, A. penicillioides, A. reticulatus,* ***A. tardicrescens****, A. vitricola, Meyerozyma guilliermondii, P. chrysogenum, Wallemia canadensis* |
| RCS 22 | front, facing glued canvas | conidia of *Alternaria* (35×10 µm), *Aspergillus*/*Penicillium* (4.0×3.3 µm; 5.5×4.0 µm, 4.3×3.6 µm), *Cladosporium* (7.5×4.5 µm), *Wallemia* (3.0×2.5 µm); mycelium | ***A. destruens****, A. magnivesiculatus, A. vitricola, A. tardicrescens, Aspergillus* sp., *P. tardochrysogenum* |
|  | front, paint layer at the torn canvas in the middle of the painting | conidia of *Aspergillus* (3.5×3.3 µm; 3.2×2.5 µm), *Cladosporium* (5.6×3.0 µm; 9.3×6.5 µm); mycelium | ***A. magnivesiculatus****, P. chrysogenum* |
|  | front, grey stains, peeling paint layer | conidia of *Aspergillus* (4.0×3.5 µm), *Cladosporium* (7.0×5.0 µm; 7.5×5.0 µm); mycelium | ***A. magnivesiculatus****, A. vitricola, A. jensenii, A. salinicola,* ***A. tardicrescens****, Bjerkandera adusta, Peniophora pithya* |
|  | back, weathered paint at the right bottom | conidia of *Aspergillus* (3.9×3.4 µm), *Cladosporium* (7.0×4.0 µm; 8.0×4.0 µm) | *A. vitricola, A. magnivesiculatus, A. destruens, A. tardicrescens, Cladosporium* sp., *Peniophora pithya, Wallemia* sp. |
|  | front, non-mouldy **control** site (cracking, peeling) | - | *A. vitricola, A. pseudoglaucus* |
| RCS 23 | front, white stains, mycelium, center: tearing, mechanical damage, color deviation at the edges | conidia of *Aspergillus* (2.5×2.0 µm), unidentified conidia (5.7×3.8 µm) | - |
|  | back, right upper margin, white mycelium | brown conidia (3×3µm; 2.5×2 µm); *Penicillium* brushes, thin long hyphae with lateral conidia | *Aspergillus* sp. |
| RCS 24 | front, center: grey stains on a dark brown background, waxed surface, folded | conidia of *Aspergillus*/*Penicillium* (4.3×4.0 µm), melanized phragmospore (23.0×6.0 µm) | ***A. tardicrescens****, A. magnivesiculatus* |
|  | front, upper part: white spots, discoloration on waxed surface | unidentified conidia (2.0×2.0 µm), dark pigmented hyphae | *A. domesticus, A. luchuensis, A. penicillioides, Botryotrichum domesticum* |
|  | back, left bottom corner | conidia of *Aspergillus*/*Penicillium* (3.3×3.2 µm), unidentified black (8.2×3.8 µm), unidentified (8.0×6.5 µm); dark pigmented mycelium | *A. vitricola,* ***A. magnivesiculatus, A. tardicrescens***, *Aspergillus* sp., |
| RCS 25 | front, center, white mycelial threats in the paint cracks, beige spots | conidia of *Aspergillus*/*Penicillium* (6.5×6.0 µm; 2.5×2.2 µm; 3.0×2.7 µm), brown (6.6×4.5µm) | ***A. destruens,*** *A. infrequens* |
|  | front, lower middle: cracked paint, white mycelial threats in the paint cracks | conidia of *Aspergillus*/*Penicillium* (4.2×3.8 µm), *Cladosporium* (11.2×5.5 µm) | ***A. destruens,*** *Meyerozyma guilliermondii* |
|  | back, upper left: dense white mycelium | conidia of *Aspergillus* (2.6×1.7 µm), *Aspergillus*/*Penicillium* (3.5×2.7 µm); *Cladosporium* (8.8×4.0 µm; 14.5×8 µm) | ***A. destruens****, A. tardicrescens, Aspergillus* sp. |
| RCS 26 | front, white mycelium between the cracks | conidia of *Aspergillus* (4.0×3.0 µm, vesicle 11.5×16 µm; 2.9 x 1.8 µm), unidentified conidia (6.5×1.5 µm), *Cladosporium* (6.5×4.0 µm); thin hyphae (1µm) | ***A. destruens*** |
|  | front, paint layer peeling off, beige stains | conidia of *Aspergillus* (4.0×3.5 µm), black transversely septate hyphae (9.0×6.0 µm) | *P. palitans* |
|  | back, bottom right | Clusters of globose cells (conidia?) (3 µm); dark mycelium | *A. destruens, A. vitricola* |
| RCS 36 | back, white spots | *Aspergillus* heads, mycelium | *Aspergillus* sp., *P. chrysogenum*, *Wallemia* sp. |
| GBJ 1 | front, left margin | conidia of *Aspergillus* (3.9×2.9; 7×6 µm) in heads | ***Cladosporium* sp*.*** (*herbarum* sp. complex), ***A. vitricola*** |
|  | front, mouldy blue pigment layer in the left quarter | conidia of *Aspergillus* conidia (6.7×5.8 µm) in heads | *A. jensenii,* ***C. westerdijkiae****, Debaryomyces hansenii, P. brevicompactum* |
| GBJ 2 | front, left edge: mouldy spots on brown and green pigments | conidia of *Aspergillus* (9.5×7.4 µm; 3.5×2.8 µm) in heads | *Alternaria* sp*., Beauveria pseudobassiana,****C. pseudocladosporioides****,* ***P. chrysogenum, P. scabrosum****,* |
|  | front, center: mouldy spots (several mm diam) on brown and green pigments | - | ***P. rubens****, P. brevicompactum,* ***Trichoderma longibrachiatum****,* ***C. westerdijkiae*** |
| GM 1 | front, dark green stains on yellow, red and blue paint | */* | *Akanthomyces muscarius, Cladosporium* sp*., Debaryomyces hansenii, P. chrysogenum* |
| GM 2 | front, small black spots in the upper part | */* | *Alternaria* sp*., Beauveria pseudobassiana,* ***Zalaria obscura,*** *P. expansum,* |
|  | front, small black spots in the right lower corner | */* | *Alternaria* sp*., C. xylophilum,* ***Debaryomyces hansenii****, P. chrysogenum,* |
| AIO-1 | mouldy front, white-greenish layers | conidia of *Aspergillus* (2.7×2.6 µm) in heads; brush-like conidiophores of *Penicillium* | *A. jensenii, A. proliferans, A. pseudoglaucus, P. chrysogenum* |
| AIO-2 | front, mouldy paint layer applied as impasto; cracks | conidia of *Aspergillus* (2.7×2.3 µm) in heads | ***A. proliferans*** |
| AIO-3 | front, mouldy paint layer applied as impasto; cracks | */* | *A. jensenii, P. chrysogenum* |
| ART-1 | front, mat surface at the bottom right part of the painting (dust accumulation?) | dematiaceous septate conidia (40×7 µm, 18×4 µm), hyaline globose conidia (6×5 µm) | *Alternaria* sp., *A. flavus, A. protuberus, A. oryzae, Arthrinium arundinis, Arthrinium marii, Botrytis cinerea, Epicoccum* sp., *C. cladosporioides, C. xylophilum, C. pseudocladosporioides, C. westerdijkiae, Curvularia coatesiae, P. corylophilum, P. palitans, P. citrinum, Porostereum* sp., *Periconia pseudobyssoides, Pseudopithomyces palmicola* |
|  | back, presumably mouldy | black hyphal fragments, unidentified single dematiaceous conidia: septate (30×4 µm), echinulate (10×5 µm), muriform (30×20 µm), oblong (3×2 µm), dematiaceous mycelium | *Alternaria rosae, A. pseudoglaucus, Bjerkandera adusta, C. pseudocladosporioides, Coprinellus micaceus, Filobasidium wieringae, Hypoxylon perforatum, P. corylophilum, P. palitans* |
|  | front, **control** site | *-* | *Alternaria* sp*., C. perangustum, C. pseudocladosporioides, C. velox, Chaetomium globosum, Cylindrobasidium* sp., *Dichotomopilus erectus, Dothiora* sp*., Neosetophoma guiyangensis, P. steckii* |
| ART-2 | front, cracked after restoration | *-* | *C. sphaerospermum, P. corylophilum, Peroneutypa scoparia, Phlebia* sp*., Stereum* sp*., Talaromyces rugulosus* |
|  | back, presumably mouldy | dematiaceous hyphal fragment | *Phlebia* sp*.* |
|  | front, **control** site | *-* | *Peniophora cinerea, A. pseudoglaucus* |
| ART-4 | front, mouldy spots | numerous particles - dust and conidia | *Alternaria sp., A. jensenii, A. magnivesiculatus, A. montevidensis A. pragensis, A. proliferans, A. pseudoglaucus, A. ruber, A. section Aspergillus, A. tardicrescens, A. vitricola, Botryotrichum murorum, Coniochaeta ligniaria, P. rubens, P. brevicompactum, P. chrysogenum, P. glabrum, P. solitum, Stereum hirsutum* |
|  | back, mouldy | conidia and hyphae of *Cladosporium* (6.5×3.0 µm), *Alternaria* (36×10 µm); *Fusarium* (?) (32.7×3.2 µm) | *Alternaria sp., A. jensenii, A. neocarnoyi, A. proliferans, A. section Aspergillus, A. sydowii, A. tardicrescens, A. vitricola, C. allicinum, C. cladosporioides, C. pseudocladosporioides, C. westerdijkiae, P. brevicompactum, P. chrysogenum, P. polonicum, P. raistrickii, P. rubens, P. solitum, Wallemia sp.* |
| ART-5 | front, mouldy spots | conidia of *Aspergillus* (4.8×4.0 µm), *Aspergillus* vesicles (12×15 µm), conidiophores (5.8 µm wide) | *Alternaria sp., A. domesticus, A. kumbius, A. niveoglaucus, A. proliferans, A. steynii,* ***A. vitricola****, Aspergillus sp., C. neolangeronii, C. pseudocladosporioides, C. westerdijkiae, P. brevicompactum,* ***P. chrysogenum****, P. olsonii* |
|  | back, mouldy | */* | *A.* section *Aspergillus, A. vitricola, A. westerdijkiae, C. pseudocladosporioides, P. olsonii* |

Legend: *A.*: *Aspergillus*; *P.*: *Penicillium*; *C.*: *Cladosporium*; -: no fungal structures observed; /: no microscopical examination;

## Supplementary Table 3. Sampling sites on the paintings, sampling methods, observed anomalies, and representative identified fungal isolates in Ex Culture Collection, and identification barcode submitted to GenBank.

| Painting code | Sampling site | Sampling method | Culture medium | Fungal species | Representative strain (EXF-) | GenBank Accession number |
| --- | --- | --- | --- | --- | --- | --- |
| RCS 15 | A, B: back, mouldy glued canvas | S, RP | DG18, MEA+5%NaCl | *Aspergillus destruens* | 7651 | benA: MW369703 |
|  |  | S | DG18 | *Aspergillus vitricola* | 7700 | benA: MW357196 |
|  |  | S | MEA | *Penicillium corylophilum* | 7650 | benA: MW357216 |
|  | C: back, mouldy white stain along the edge | S, RP | MEA+5%NaCl, DG18 | *Aspergillus jensenii* | 7652 | benA: MW357106 |
|  |  | S | MEA | *Penicillium rubens* | 7653 | benA: MW357085 |
|  | D: back, cobweb along the left margin | S | DG18 | *Aspergillus destruens* | 10407 | benA: MW369698 |
|  |  | S | DRBC | *Aspergillus creber* | 10410 | benA: MW357100 |
|  |  | S | HA+5%NaCl | *Aspergillus fumigatus* | 10413 | benA: MW357177 |
|  |  | S | MY50G, DG18, HA+5%NaCl | *Aspergillus jensenii* | 10405 | benA: MW357112 |
|  |  | S | MY10-12 | *Aspergillus* sp*.* | 10406 | - |
|  |  | S | M9 | *Debaryomyces hansenii* | 10338 | ITS: MW288760 |
|  |  | S | DG18 | *Penicillium* sp. | 10408 | - |
|  | E: front, mouldy paint | S | MY10-12, MEA, MY50G | *Aspergillus destruens* | 10211 | benA: MW369688 |
|  |  | S | MY10-12 | *Wallemia* aff. *muriae* | 10201 | ITS: MW288060 |
|  | F: front, control (not mouldy) | S | MY50G | *Aspergillus destruens* | 10582 | benA: MW369692 |
|  |  | S | DG18 | *Aspergillus domesticus* | 10583 | benA: MW357171 |
|  |  | S | MY50G | *Aspergillus vitricola* | 10585 | - |
|  |  | S | DG18 | *Penicillium bialowiezense* | 10580 | benA: MW357227 |
| RCS 16 | A: back, mouldy green stain at the glued canvas | S | MEA | *Aspergillus creber* | 7659 | benA: MW357093 |
|  |  | S | MEA+5%NaCl, DG18 | *Aspergillus destruens* | 7661 | benA: MW369708 |
|  |  | S | DG18 | *Aspergillus sloanii* | 7656 | benA: MW357182 |
|  |  | S | MEA | *Chaetomium subaffine* | 7658 | ITS: MW288930 |
|  |  | S | MEA | *Penicillium corylophilum* | 7655 | benA: MW357217 |
|  | B: back, white mouldy stain | S | MEA+5%NaCl | *Aspergillus conicus* | 7660 | benA: MW357091 |
| RCS 17 | Front: grey powdery paint layer in the right lower corner | S | MEA+5%NaCl, DG18 | *Aspergillus destruens* | 7665 | benA: MW369704 |
|  |  | S | MEA+5%NaCl | *Aspergillus conicus* | 7663 | benA: MW357092 |
|  |  | S | MEA | *Aspergillus puulaauensis* | 7668 | benA: MW357119 |
|  |  | S | MEA+5%NaCl | *Wallemia* aff. *muriae* | 7664 | ITS: MW288061 |
| RCS 18 | A: back, grey stain at the bottom margin | S | MEA, DG18 | *Chaetomium cochliodes* | 7669 | ITS: MW288932 |
|  |  | S | MEA | *Chaetomium* sp. | 7670 | ITS: MW288934 |
|  | B: back, black stain at the thinned canvas | S | MEA | *Chaetomium cochliodes* | 7690 | ITS: MW288933 |
|  |  | S | MEA | *Botryotrichum domesticum* | 7692 | ITS: MW288938 |
| RCS 19 | A: wooden frame with white fungal spots | S | MEA, DG18 | *Aspergillus jensenii* | 7672 | benA: MW357103 |
|  |  | S | MY10-12 | *Wallemia* aff. *muriae* | 7671 | ITS: MW288062 |
|  | B: back (canvas) at the bottom left corner | S | DG18, MEA | *Aspergillus creber* | 7676 | benA: MW357094 |
|  |  | S | MEA+5%NaCl | *Aspergillus puulaauensis* | 7678 | benA: MW357120 |
|  |  | S | MY10-12, MEA+5%NaCl | *Wallemia canadensis* | 7674 | ITS: MW288070 |
| RCS 20 | A: front, gray stains of 2-20 mm diameter on brown stained and cracked paint layer | S | MY50G | *Aspergillus* sp. | 10389 | - |
|  |  | S | MY10-12, DG18, MY50G | *Wallemia* aff. *muriae* | 10120 | ITS: MW288065 |
|  | B: back, upper right, white threads | S | MY10-12, DG18, MY50G | *Aspergillus vitricola* | 10199 | benA: MW357203 |
|  |  | S | DG18 | *Wallemia* aff. *muriae* | 10122 | ITS: MW288067 |
| RCS 21 | A: front, cracked and flaking paint layer with grey stains in the bottom right corner; peeling paint layer | S | DG18, MY50G | *Aspergillus magnivesiculatus* | 10377 | benA: MW357158 |
|  |  | S | DG18 | *Aspergillus reticulatus* | 10339 | benA: MW357167 |
|  |  | S | MY10-12, MY50G | *Aspergillus tardicrescens* | 10340 | benA: MW357131 |
|  |  | S | MY50G | *Aspergillus vitricola* | 10378 | - |
|  |  | S | MY50G | *Aspergillus glaucus* | 10423 | benA: MW357178 |
|  | B: back, grey-white spots at the bottom | S | MY10-12 | *Aspergillus creber* | 10375 | benA: MW357098 |
|  |  | S | MY50G | *Aspergillus domesticus* | 10384 | benA: MW357174 |
|  |  | S | DG18 | *Aspergillus magnivesiculatus* | 10380 | benA: MW357147 |
|  |  | S | DG18 | *Aspergillus penicillioides* | 10422 | benA: MW357161 |
|  |  | S | DG18, MY50G | *Aspergillus reticulatus* | 10381 | benA: MW357170 |
|  |  | S | MY10-12, MY50G | *Aspergillus tardicrescens* | 10382 | benA: MW357132 |
|  |  | S | DG18, MY50G | *Aspergillus vitricola* | 10376 | benA: MW357197 |
|  |  | S | MY10-12 | *Meyerozyma guilliermondii* | 10452 | ITS: MW288737 |
|  |  | S | DG18 | *Penicillium chrysogenum* | 10451 | benA: MW357235 |
|  |  | S | MY10-12 | *Wallemia canadensis* | 10342 | ITS: MW288071 |
| RCS 22 | A: front, facing glued canvas | S | DG18 | *Aspergillus destruens* | 10379 | benA: MW369709 |
|  |  | S | DG18 | *Aspergillus magnivesiculatus* | 10387 | benA: MW357157 |
|  |  | S | MY10-12 | *Aspergillus vitricola* | 10427 | - |
|  |  | S | DG18, MY10-12 | *Aspergillus tardicrescens* | 10453 | benA: MW357144 |
|  |  | S | DG18 | *Aspergillus* sp. | 10419 | - |
|  |  | S | MY50G | *Penicillium tardochrysogenum* | 10343 | benA: MW357090 |
|  | B: front, paint layer at the torn canvas in the middle of the painting | S | MY50G | *Aspergillus magnivesiculatus* | 10344 | benA: MW357155 |
|  |  | S | MY50G | *Penicillium chrysogenum* | 10430 | ITS: OQ410483 |
|  | C: front, grey stains, peeling off the paint layer | S | DG18, MY50G, MY10-12 | *Aspergillus magnivesiculatus* | 10345 | benA: MW357151 |
|  |  | S | DG18, MY50G | *Aspergillus vitricola* | 10400 | benA: MW357188 |
|  |  | S | DRBC, MY50G | *Aspergillus jensenii* | 15112 | benA: MW357118 |
|  |  | S | DG18 | *Aspergillus salinicola* | 10401 | benA: MW357181 |
|  |  | S | DG18, MY10-12 | *Aspergillus tardicrescens* | 10402 | benA: MW357138 |
|  |  | S | DRBC | *Bjerkandera adusta* | 15111 | ITS: MW288722 |
|  |  | S | DRBC | *Peniophora pithya* | 15105 | ITS: MW288740 |
|  | D: back, weathered paint at the right bottom | C | DG18, MY50G | *Aspergillus vitricola* | 15122 | benA: MW357202 |
|  |  | S | DG18 | *Aspergillus magnivesiculatus* | 15126 | benA: MW357159 |
|  |  | S | DG18 | *Aspergillus destruens* | 10349 | benA: MW369713 |
|  |  | S | DG18, MY50G, MY10-12 | *Aspergillus tardicrescens* | 10351 | benA: MW357133 |
|  |  | S | MY50G | *Cladosporium* sp. | 10397 | ITS: MW289525 |
|  |  | C | DG18 | *Peniophora pithya* | 15121 | ITS: MW288742 |
|  |  | S | MY10-12, MY50G | *Wallemia* sp. | 10348 | - |
|  | 2A: front, non-mouldy control site (cracking, peeling) | S, C | DG18, MY50G | *Aspergillus vitricola* | 15114 | benA: MW357209 |
|  |  | C | DG18 | ***Aspergillus pseudoglaucus*** | **15119** | benA: MW357166 |
| RCS 23 | D: back, right upper margin, white mycelium | S | MY50G | *Aspergillus* sp. | 10389 | - |
| RCS 24 | A: front, center: gray stains on a dark brown background, waxed surface, folded appearance | S | DG18, MY50G | *Aspergillus tardicrescens* | 10352 | benA: MW357134 |
|  |  | S | MY10-12, DG18 | *Aspergillus magnivesiculatus* | 10353 | benA: MW357145 |
|  | B: front, upper part (upper angel): whitish spots, discoloration on waxed surface | S | MY10-12, M9 | *Aspergillus domesticus* | 10424 | benA: MW357173 |
|  |  | S | MY10-12 | *Aspergillus luchuensis* | 10394 | benA: MW357180 |
|  |  | S | MY50G, MY10-12 | *Aspergillus penicillioides* | 10355 | benA: MW357163 |
|  |  | S | M9 | *Botryotrichum domesticum* | 10840 | ITS: MW288937 |
|  | C: back, left bottom corner (no data) | S | MY10-12 | *Aspergillus* sp. | 10459 | - |
|  |  | S | DG18 | *Aspergillus vitricola* | 10461 | benA: MW357198 |
|  |  | S | MY50G, DG18 | *Aspergillus magnivesiculatus* | 10356 | benA: MW357146 |
|  |  | S | MY10-12 | *Aspergillus tardicrescens* | 10358 | benA: MW357141 |
| RCS 25 | A: front, center, white mycelial threats in the paint cracks, beige spots | S | **MY10-12, MY50G, DG18** | ***Aspergillus destruens*** | 10359 | benA: MW369691 |
|  |  | S | HA5%NaCl | *Aspergillus infrequens* | 10450 | benA: MW357179 |
|  | B: front, lower middle: cracked paint, white mycelial threats in the paint cracks | S | MY10-12 | *Meyerozyma guilliermondii* | 10457 | ITS: MW288736 |
|  | C: back, upper left: dense white mycelium | S | **MY50G, MY10-12, HA5%NaCl** | ***Aspergillus destruens*** | 10391 | benA: MW369694 |
|  |  | S | HA5%NaCl | *Aspergillus tardicrescens* | 10431 | benA: MW357143 |
|  |  | S | DG18 | *Aspergillus* sp. | 10366 | ITS: MW288767 |
| RCS 26 | A: front, white mycelium between the cracks | S | MY50G | *Aspergillus destruens* | 10367 | benA: MW369697 |
|  | B: front, paint layer peeling off, beige stains | S | MY50G | *Penicillium palitans* | 10368 | benA: MW357080 |
|  | C: back, bottom right (no data) | S | DG18, MY12-12, MY50G | *Aspergillus destruens* | 10411 | benA: MW369705 |
|  |  | S | MY10-12, MY50G | *Aspergillus vitricola* | 10463 | benA: MW357199 |
| GBJ 1 | A: front, left margin next to the frame | S | **MY50G, DRBC, MEA; MY10-12, DG18, HA** | ***Cladosporium* sp*.* (*herbarum* sp. complex)** | 10552 | ITS: MW289546 |
|  |  | S | **MY50G, DG18, MY10-12** | ***Aspergillus vitricola*** | 10568 | benA: MW357190 |
|  | B: front, mouldy blue pigment layer in the left quarter | S | MEA | *Debaryomyces hansenii* | 10482 | ITS: MW288761 |
|  |  | S | MEA, DRBC | *Penicillium brevicompactum* | 10484 | benA: MW357229 |
|  | C: front, mouldy blue pigment layer in the left quarter | S(d) | DG18, MY50G | *Aspergillus jensenii* | 10491 | benA: MW357108 |
| GBJ 2 | A: front, left edge beside the frame: mouldy spots (several mm diam) on brown and green pigments | S | MY10-12, NA, MY50G | *Alternaria* sp*.* | 10494 | ITS: MW288709 |
|  |  | S | NA | *Beauveria pseudobassiana* | 10556 | ITS: MW288719 |
|  |  | S | MY50G, DG18, MY10-12, MEA | *Cladosporium pseudocladosporioides* | 10558 | TEF: MW387149 |
|  |  | S | DG18 | *Penicillium chrysogenum* | 10495 | benA: MW357233 |
|  |  | S | DG18 | *Penicillium scabrosum* | 10557 | benA: MW357087 |
|  |  | S | MY10-12 | *Penicillium chrysogenum* | 10493 | benA: MW357078 |
|  | B: front, center: mouldy spots (several mm diam) on brown and green pigments | S | DRBC, NA | *Trichoderma longibrachiatum* | 10501 | ITS: MW288750 |
|  | C: front, right edge: mouldy spots (several mm diam) on brown and green pigments | S | MY50G, MEA | *Penicillium rubens* | 10560 | benA: MW357084 |
|  |  | S | DRBC | *Penicillium brevicompactum* | 10506 | benA: MW357232 |
| GM 1 | front, dark green stains on yellow, red and blue paint | S | NA | *Akanthomyces muscarius* | 10648 | ITS: MW288713 |
|  |  | S | DRBC | *Cladosporium* sp*.* | 10646 | ITS: MW289545 |
|  |  | S | NA | *Debaryomyces hansenii* | 10647 | ITS: MW288762 |
|  |  | S | MY50G | *Penicillium chrysogenum* | 10669 | benA: MW357234 |
| GM 2 | front, numerous small black spots | S | NA | *Alternaria* sp*.* | 10651 | ITS: MW288712 |
|  |  | S | M9+chx | *Beauveria pseudobassiana* | 10670 | ITS: MW288721 |
|  |  | S | DRBC, MY50G | *Debaryomyces hansenii* | 10652 | ITS: MW288763 |
|  |  | S | NA+chx | *Cladosporium xylophilum* | 10656 | TEF: MW387151 |
|  |  | S | NA+5%NaCl | *Penicillium chrysogenum* | 10655 | ITS: MW288758; benA: OQ420418 |
|  |  | S | DRBC | *Penicillium expansum* | 10649 | benA: MW357226 |
|  |  | S | MY50G, NA+chx | *Zalaria obscura* | 10650 | ITS: MW288752 |
| RCS36 | back: white spots | S | MY50G | *Aspergillus* sp*.* | 11363 | ITS: MW288769 |
|  |  | S | MY50G | *Penicillium chrysogenum* | 11364 | benA: MW357236 |
|  |  | S | MY10-12 | *Wallemia* sp*.* | 11362 | ITS: MW288072 |
| AIO 1 | front, mouldy over the entire surface in the form of cobwebs and white-greenish layers | S | MEA; DG18 | *Aspergillus jensenii* | 10218 | benA: MW488410 |
|  |  | S | MY50G | *Aspergillus proliferans* | 10220 | benA: MW488414 |
|  |  | S | MY10-12 | *Aspergillus pseudoglaucus* | 10222 | benA: MW488413 |
|  |  | RP | DG18 | *Penicillium chrysogenum* | 10111 | benA: MW488404 |
| AIO 2 | front, mouldy paint layer applied as relief; local cracks | S | MY50G, MY10-12 | *Aspergillus proliferans* | 10217 | benA: MW488416 |
| AIO 3 | front, mouldy paint layer applied as relief; local cracks | RP | DG18 | *Aspergillus jensenii* | 10224 | benA: MW488412 |
|  |  | RP | DG18 | *Penicillium chrysogenum* | 10113 | benA: MW488406 |
| ART 1 | 1A: front, matte surface at the bottom right part of the painting (dust accumulation?) | S, C | DRBC, MY50G, DG18 | *Alternaria* sp. | 15054 | ITS: MW288705 |
|  |  | S | DG18 | *Aspergillus flavus* | 15071 | benA: MW357176 |
|  |  | C | MY50G | *Aspergillus protuberus* | 15063 | benA: MW357125 |
|  |  | S | DRBC | *Aspergillus oryzae* | 15068 | benA: MW357160 |
|  |  | C | DRBC, MY50G | *Arthrinium arundinis* | 15057 | ITS: MW288714 |
|  |  | C | MY50G | *Arthrinium marii* | 15082 | ITS: MW288716 |
|  |  | C | DRBC | *Botrytis cinerea* | 15056 | ITS: MW288724 |
|  |  | C | DRBC, DG18 | *Epicoccum* sp. | 15055 | ITS: MW288732 |
|  |  | S, C | DG18 | *Cladosporium cladosporioides* | 15062 | Act: MW387136 |
|  |  | S | DRBC | *Cladosporium xylophilum* | 15070 | Act: MW387137 |
|  |  | S, C | DG18 | *Cladosporium pseudocladosporioides* | 15077 | Act: MW369719 |
|  |  | S | DG18 | *Cladosporium westerdijkiae* | 15073 | Act: MW369727 |
|  |  | S | DRBC | *Curvularia coatesiae* | 15076 | ITS: MW288726 |
|  |  | S | DRBC, DG18, MY50G | *Penicillium corylophilum* | 15081 | benA: MW357224 |
|  |  | S | DRBC, MY50G | *Penicillium palitans* | 15066 | benA: MW357082 |
|  |  | S | DG18 | *Penicillium citrinum* | 15078 | benA: MW357212 |
|  |  | C | DRBC | *Porostereum* sp. | 15058 | ITS: MW288746 |
|  |  | S | DG18 | *Periconia pseudobyssoides* | 15072 | ITS: MW288743 |
|  |  | C | DRBC | *Pseudopithomyces palmicola* | 15074 | ITS: OQ410482 |
|  | 2A: front, control site without fungal contamination | S | MY50G | *Alternaria* sp*.* | 15092 | ITS: MW288708 |
|  |  | C | MY50G | *Cladosporium perangustum* | 15087 | Act: MW387138 |
|  |  | S | MY50G | *Cladosporium pseudocladosporioides* | 15091 | Act: MW369720 |
|  |  | C | DRBC | *Cladosporium velox* | 15085 | benA: MW369716 |
|  |  | S | DRBC | *Chaetomium globosum* | 15090 | ITS: MW288935 |
|  |  | C | DRBC | *Cylindrobasidium* sp*.* | 15086 | ITS: MW288728 |
|  |  | C | DRBC | *Dichotomopilus erectus* | 15084 | ITS: MW288731 |
|  |  | C | MY50G | *Dothiora* sp*.* | 15088 | ITS: MW288730 |
|  |  | C | DRBC | *Neosetophoma guiyangensis* | 15083 | ITS: MW288738 |
|  |  | S | DRBC | *Penicillium steckii* | 15089 | benA: MW357089 |
|  | 3A: back, presumably mouldy | S | DRBC | *Alternaria rosae* | 15097 | ITS: MW288704 |
|  |  | S | DG18 | *Aspergillus pseudoglaucus* | 15102 | benA: MW357165 |
|  |  | C | DRBC | *Bjerkandera adusta* | 15093 | ITS: MW288723 |
|  |  | S | DRBC, DG18 | *Cladosporium pseudocladosporioides* | 15098 | Act: MW369721 |
|  |  | C | DRBC | *Coprinellus micaceus* | 15213 | ITS: MW288725 |
|  |  | S | DRBC | *Filobasidium wieringae* | 15100 | ITS: MW288734 |
|  |  | C | DG18 | *Hypoxylon perforatum* | 15095 | ITS: MW288735 |
|  |  | S, C | DRBC, MY50G | *Penicillium corylophilum* | 15094 | benA: MW357221 |
|  |  | S | DRBC | *Penicillium palitans* | 15096 | benA: MW357081 |
| ART 2 | 1A: front, the top layer of the painting cracked after restoration | C | DG18 | *Cladosporium sphaerospermum* | 15133 | Act: MW387139 |
|  |  | C | DG18 | *Penicillium corylophilum* | 15132 | benA: MW357215 |
|  |  | S | DRBC | *Peroneutypa scoparia* | 15134 | ITS: MW288744 |
|  |  | C | DRBC | *Phlebia* sp*.* | 15165 | ITS: MW288745 |
|  |  | C | DRBC | *Stereum* sp*.* | 15131 | ITS: MW288748 |
|  |  | S | DG18 | *Talaromyces* sp. | 15138 | - |
|  | 2A: front, control site without fungal contamination | C | DRBC | *Peniophora cinerea* | 15135 | ITS: MW288741 |
|  |  | C | DG18 | *Aspergillus pseudoglaucus* | 15136 | benA: MW357164 |
|  | 3A: back, presumably mouldy | C | DRBC | *Phlebia* sp*.* | 15137 | ITS: MW288739 |
| ART 4 | 1A: front, mouldy spots | C | **DG18, MY50G** | ***Aspergillus jensenii*** | 15474 | benA: OQ446684 |
|  |  | C | **DG18, MY50G** | ***Aspergillus vitricola*** | 15473 | benA: OQ446675 |
|  |  | C | MY50G | *Aspergillus montevidensis* | 15519 | benA: OQ446665 |
|  |  | C | DG18 | *Aspergillus proliferans* | 15478 | benA: OQ446687 |
|  |  | C | DG18, MY50G | *Aspergillus pragensis* | 15513 | benA: OQ446690 |
|  |  | C | DG18, MY50G | *Aspergillus pseudoglaucus* | 15511 | benA: OQ446664 |
|  |  | C | MY50G | *Aspergillus ruber* | 15521 | benA: OQ446666 |
|  |  | C | DG18 | *Aspergillus* section *Aspergillus* | 15480 | - |
|  |  | C | DRBC | *Coniochaeta ligniaria* | 15505 | ITS: OR097691 |
|  |  | C | **DG18, MY50G** | ***Penicillium brevicompactum*** | 15479 | benA: OQ420408 |
|  |  | C | DRBC, MY50G | *Penicillium rubens* | 15472 | benA: OQ420406 |
|  |  | C | **DRBC, DG18, MY50G** | ***Penicillium chrysogenum*** | 15475 | benA: OQ420412 |
|  |  | C | DG18 | *Penicillium glabrum* | 15510 | benA: OQ420419 |
|  | 1D: front, mouldy spots | S | MY50G | *Aspergillus magnivesiculatus* | 15500 | benA: OQ446663 |
|  |  | S | DRBC | *Aspergillus pragensis* | 15522 | benA: OQ446691 |
|  |  | S | **DRBC, DG18, MY50G** | ***Aspergillus jensenii*** | 15523 | benA: OQ446685 |
|  |  | S | **DG18, MY50G** | ***Aspergillus vitricola*** | 15524 | benA: OQ446677 |
|  |  | S | DG18, MY50G | *Aspergillus tardicrescens* | 15491 | benA: OQ410478 |
|  |  | S | DG18 | *Alternaria* sp*.* | 15495 | ITS: OQ410479 |
|  |  | S | DRBC | *Botryotrichum murorum* | 15485 | ITS: OQ410476 |
|  |  | S | **DG18** | ***Penicillium brevicompactum*** | 15488 | - |
|  |  | S | **DRBC, DG18** | ***Penicillium chrysogenum*** | 15486 | benA: OQ420413 |
|  |  | S | DG18 | *Penicillium solitum* | 15526 | benA: OQ420420 |
|  |  | S | DG18 | *Stereum hirsutum* | 15528 | ITS: OQ410480 |
|  | 3A: back, presumably mouldy | C | DG18 | *Aspergillus sydowii* | 15542 | benA: OQ446667 |
|  |  | C | **DG18** | ***Aspergillus jensenii*** | 15544 | benA: OQ446686 |
|  |  | C | MY50G | *Aspergillus tardicrescens* | 15549 | benA: OQ446668 |
|  |  | C | MY50G | *Aspergillus proliferans* | 15550 | benA: OQ446688 |
|  |  | C | MY50G | *Aspergillus neocarnoyi* | 15551 | benA: OQ446669 |
|  |  | C | MY50G | *Aspergillus vitricola* | 15632 | benA: OQ446678 |
|  |  | C | DG18, MY50G | *Aspergillus* section *Aspergillus* | 15558 | - |
|  |  | C | DRBC | *Penicillium polonicum* | 15536 | benA: OQ420421 |
|  |  | C | **DG18** | ***Penicillium brevicompactum*** | 15537 | benA: OQ420410 |
|  |  | C | **DG18, MY50G** | ***Penicillium rubens*** | 15541 | benA: OQ420407 |
|  |  | C | DG18 | *Penicillium chrysogenum* | 15548 | benA: OQ420415 |
|  |  | C | **DG18, MY50G** | ***Cladosporium pseudocladosporioides*** | 15538 | Act: OR102450 |
|  |  | C | DG18 | *Wallemia s*p. | 15631 | - |
|  | 3D: back, presumably mouldy | S | **DRBC, DG18, MY50G** | ***Aspergillus jensenii*** | 15561 | benA: OQ446683 |
|  |  | S | DG18, MY50G | *Aspergillus vitricola* | 15569 | benA: OQ446679 |
|  |  | S | DG18 | *Alternaria* sp*.* | 15566 | - |
|  |  | S | **DRBC, DG18** | ***Penicillium brevicompactum*** | 15562 | benA: OR102447 |
|  |  | S | **DRBC, DG18** | ***Penicillium rubens*** | 15559 | benA: OR102446 |
|  |  | S | DG18 | *Penicillium solitum* | 15564 | benA: OR102448 |
|  |  | S | MY50G | *Penicillium raistrickii* | 15570 | benA: OR102449 |
|  |  | S | **DG18, MY50G** | ***Cladosporium pseudocladosporioides*** | 15571 | Act: OR102453 |
|  |  | S | DRBC | *unidentified* | 15560 | - |
| ART-5 | 1A, 2A: front, mouldy spots | C | DRBC, DG18 | *Aspergillus kumbius* | 15575 | benA: OQ446670 |
|  |  | C | **DG18, MY50G** | ***Aspergillus vitricola*** | 15649 | benA: OQ446680 |
|  |  | C | DG18 | *Aspergillus proliferans* | 15590 | benA: OQ446689 |
|  |  | C | MY50G | *Aspergillus niveoglaucus* | 15592 | benA: OQ446671 |
|  |  | C | DG18 | *Aspergillus* sp*.* | 15577 | - |
|  |  | C | DRBC | *Alternaria* sp*.* | 15584 | ITS: OQ410481 |
|  |  | C | DRBC, DG18 | *Cladosporium neolangeronii* | 15574 | Act: OR102458 |
|  |  | C | DRBC, DG18 | *Cladosporium westerdijkiae* | 15586 | Act: OR102457 |
|  |  | C | **DG18** | ***Penicillium chrysogenum*** | 15591 | benA: OQ420416 |
|  | 1D, 2D: front, mouldy spots | S | DG18 | *Aspergillus domesticus* | 15637 | benA: OQ446674 |
|  |  | S | MY50G | *Aspergillus steynii* | 15582 | benA: OQ446673 |
|  |  | S | **DG18, MY50G** | ***Aspergillus vitricola*** | 15579 | benA: OQ446681 |
|  |  | S | MY50G | *Aspergillus* sp*.* | 15661 | - |
|  |  | S | DRBC | *Cladosporium pseudocladosporioides* | 15594 | Act: OR102451 |
|  |  | S | MY50G | *Penicillium brevicompactum* | 15583 | benA: OQ420411 |
|  |  | S | **DG18** | ***Penicillium chrysogenum*** | 15595 | benA: OQ420417 |
|  |  | S | DRBC | *Penicillium olsonii* | 15593 | benA: OQ420422 |
|  | 3D: back, presumably mouldy | S | **DG18, MY50G** | ***Aspergillus vitricola*** | 15607 | benA: OQ446682 |
|  |  | S | DG18, MY50G | *Aspergillus* section *Aspergillus* | 15604 | - |
|  |  | S | DRBC | *Aspergillus westerdijkiae* | 15599 | benA: OQ446672 |
|  |  | S | DG18, MY50G | *Cladosporium pseudocladosporioides* | 15603 | Act: OR102452 |
|  |  | S | DRBC, DG18 | *Penicillium olsonii* | 15600 | benA OQ420423 |

Legend: S: cotton swab dipped in physiological solution; S(d): dry cotton swab; C: Copan swab; RP: RODAC plate print. -: sequence not deposited (morphological identification only).

## Supplementary Table 4. Isolated fungal species on culture media.

| Species affili-ation | culture medium with water activity (a_w_) | **DRBC a_w_ 0.997** | **MEA a_w_ 0.99** | **MEA+5%NaCl a_w_ 0.965** | **DG18 a_w_ 0.955** | **MY50G a_w_ 0.89** | **MY10-12 a_w_ 0.88** | **other media** |
| --- | --- | --- | --- | --- | --- | --- | --- | --- |
|  | *number of sampled paintings from which fungi were isolated* | 10 | 8 | 4 | 19 | 17 | 13 | 1 - 2 |
|  | *number of isolated species* | 47 | 14 | 6 | 46 | 35 | 17 | 1 - 3 |
| *A* | ***Alternaria*** *rosae* | X |  |  |  |  |  |  |
| *A* | *Alternaria* sp. | X |  |  | X | X | X |  |
| *A* | *Alternaria* sp. | X |  |  |  |  |  |  |
| *A* | ***Arthrinium*** *arundinis* | X |  |  |  | X |  |  |
| *A* | *Arthrinium marii* |  |  |  |  | X |  |  |
| *A* | ***Aspergillus*** *conicus* ^(sect.^ *^Restricti^*^)^ |  |  | X |  |  |  |  |
| *A* | *Aspergillus creber* | X | X |  | X |  | X |  |
| *A* | *Aspergillus destruens* ^(sect.^ *^Restricti^*^)^ |  | X | X | X | X | X |  |
| *A* | *Aspergillus domesticus* ^(sect.^ *^Restricti^*^)^ |  |  |  | X | X | X | M9 |
| *A* | *Aspergillus flavus* |  |  |  | X |  |  |  |
| *A* | *Aspergillus fumigatus* |  |  |  |  |  |  | NA+5%NaCl |
| *A* | *Aspergillus glaucus* |  |  |  |  | X |  |  |
| *A* | *Aspergillus jensenii* | X | X | X | X | X |  | NA+5%NaCl |
| *A* | *Aspergillus kumbius* | X |  |  | X |  |  |  |
| *A* | *Aspergillus luchuensis* ^(sect.^ *^Aspergillus^*^)^ |  |  |  |  |  | X |  |
| *A* | *Aspergillus magnivesiculatus* ^(sect.^ *^Restricti^*^)^ |  |  |  | X | X | X |  |
| *A* | *Aspergillus neocarnoyi* ^(sect.^ *^Aspergillus^*^)^ |  |  |  |  | X |  |  |
| *A* | *Aspergillus niveoglaucus* |  |  |  |  | X |  |  |
| *A* | *Aspergillus oryzae* | X |  |  |  |  |  |  |
| *A* | *Aspergillus penicillioides* ^(sect.^ *^Restricti^*^)^ |  |  |  | X | X | X |  |
| *A* | *Aspergillus pragensis* | X |  |  | X | X |  |  |
| *A* | *Aspergillus proliferans^.^* ^(sect.^ *^Restricti^*^)^ |  |  |  | X | X | X |  |
| *A* | *Aspergillus protuberus* ^(sect.^ *^Aspergillus^*^)^ |  |  |  |  | X |  |  |
| *A* | *Aspergillus pseudoglaucus* ^(sect.^ *^Aspergillus^*^)^ |  |  |  | X | X | X |  |
| *A* | *Aspergillus puulaauensis* |  | X | X |  |  |  |  |
| *A* | *Aspergillus reticulatus* ^(sect.^ *^Restricti^*^)^ |  |  |  | X | X |  |  |
| *A* | *Aspergillus ruber* |  |  |  |  | X |  |  |
| *A* | *Aspergillus salinicola* ^(sect.^ *^Restricti^*^)^ |  |  |  | X |  |  |  |
| *A* | *Aspergillus* sp. ^(sect.^ *^Aspergillus^*^)^ |  |  |  | X | X |  |  |
| *A* | *Aspergillus sloanii* ^(sect.^ *^Aspergillus^*^)^ |  |  |  | X |  |  |  |
| *A* | *Aspergillus* sp. ^(sect.^ *^Aspergillus^*^)^ |  |  |  | X | X | X |  |
| *A* | *Aspergillus steynii* ^(sect.^ *^Aspergillus^*^)^ |  |  |  |  | X |  |  |
| *A* | *Aspergillus sydowii* |  |  |  | X |  |  |  |
| *A* | *Aspergillus tardicrescens* ^(sect.^ *^Restricti^*^)^ |  |  |  | X | X | X |  |
| *A* | *Aspergillus vitricola* ^(sect.^ *^Restricti^*^)^ |  |  |  | X | X | X |  |
| *A* | *Aspergillus westerdijkiae* | X |  |  |  |  |  |  |
| *A* | ***Beauveria*** *pseudobassiana* |  |  |  |  |  |  | M9+chx |
| *B* | ***Bjerkandera*** *adusta* | X |  |  |  |  |  |  |
| *A* | ***Botryotrichum*** *domesticum* |  | X |  |  |  |  | M9 |
| *A* | *Botryotrichum murorum* | X |  |  |  |  |  |  |
| *A* | ***Botrytis*** *cinerea* | X |  |  |  |  |  |  |
| *A* | ***Chaetomium*** *cochliodes* |  | X |  | X |  |  |  |
| *A* | *Chaetomium globosum* | X |  |  |  |  |  |  |
| *A* | *Chaetomium sp.* |  | X |  |  |  |  |  |
| *A* | *Chaetomium subaffine* |  | X |  |  |  |  |  |
| *A* | ***Cladosporium*** *perangustum* |  |  |  |  | X |  |  |
| *A* | *Cladosporium cladosporioides* |  |  |  | X |  |  |  |
| *A* | *Cladosporium neolangeronii* | X |  |  | X |  |  |  |
| *A* | *Cladosporium pseudocladosporioides* | X | X |  | X | X |  |  |
| *A* | *Cladosporium* sp. | X |  |  |  | X |  |  |
| *A* | *Cladosporium* sp. (herbarum sp. complex) | X | X |  | X |  |  |  |
| *A* | *Cladosporium sphaerospermum* |  |  |  | X |  |  |  |
| *A* | *Cladosporium velox* | X |  |  |  |  |  |  |
| *A* | *Cladosporium westerdijkiae* | X |  |  | X |  |  |  |
| *A* | *Cladosporium xylophilum* | X |  |  |  |  |  |  |
| *A* | ***Coniochaeta*** *ligniaria* | X |  |  |  |  |  |  |
| *B* | ***Coprinellus*** *micaceus* | X |  |  |  |  |  |  |
| *A* | ***Curvularia*** *coatesiae* | X |  |  |  |  |  |  |
| *B* | ***Cylindrobasidium*** *sp.* | X |  |  |  |  |  |  |
| *A* | ***Debaryomyces*** *hansenii* | X | X |  |  | X |  | M9 |
| *A* | ***Dichotomopilus*** *erectus* | X |  |  |  |  |  |  |
| *A* | ***Dothiora*** *sp.* |  |  |  |  | X |  |  |
| *A* | ***Epicoccum*** *sp.* | X |  |  | X |  |  |  |
| *B* | ***Filobasidium*** *wieringae* | X |  |  |  |  |  |  |
| *A* | ***Hypoxylon*** *perforatum* |  |  |  | X |  |  |  |
| *A* | ***Meyerozyma*** *guilliermondii* |  |  |  |  |  | X |  |
| *A* | ***Neosetophoma*** *guiyangensis* | X |  |  |  |  |  |  |
| *A* | ***Penicillium*** *bialowiezense* |  |  |  | X |  |  |  |
| *A* | *Penicillium brevicompactum* | X | X |  | X | X |  |  |
| *A* | *Penicillium chrysogenum* | X |  |  | X | X | X |  |
| *A* | *Penicillium citrinum* |  |  |  | X |  |  |  |
| *A* | *Penicillium corylophilum* | X | X |  | X | X |  |  |
| *A* | *Penicillium expansum* | X |  |  |  |  |  |  |
| *A* | *Penicillium glabrum* |  |  |  | X |  |  |  |
| *A* | *Penicillium olsonii* | X |  |  | X |  |  |  |
| *A* | *Penicillium palitans* | X |  |  |  | X |  |  |
| *A* | *Penicillium polonicum* | X |  |  |  |  |  |  |
| *A* | *Penicillium raistrickii* |  |  |  |  | X |  |  |
| *A* | *Penicillium rubens* | X | X |  | X | X |  |  |
| *A* | *Penicillium scabrosum* |  |  |  | X |  |  |  |
| *A* | *Penicillium solitum* |  |  |  | X |  |  |  |
| *A* | *Penicillium sp.* |  |  |  | X |  |  |  |
| *A* | *Penicillium steckii* | X |  |  |  |  |  |  |
| *B* | ***Peniophora*** *cinerea* | X |  |  |  |  |  |  |
| *B* | *Peniophora pithya* | X |  |  | X |  |  |  |
| *A* | ***Periconia*** *pseudobyssoides* |  |  |  | X |  |  |  |
| *A* | ***Peroneutypa*** *scoparia* | X |  |  |  |  |  |  |
| *B* | ***Phlebia*** *sp.* | X |  |  |  |  |  |  |
| *B* | ***Porostereum*** *sp.* | X |  |  |  |  |  |  |
| *A* | ***Pseudopithomyces*** *palmicola* | X |  |  |  |  |  |  |
| *B* | ***Stereum*** *hirsutum* |  |  |  | X |  |  |  |
| *B* | *Stereum sp.* | X |  |  |  |  |  |  |
| *A* | ***Talaromyces*** *rugulosus* |  |  |  | X |  |  |  |
| *A* | ***Trichoderma*** *longibrachiatum* | X |  |  |  |  |  |  |
| *B* | ***Wallemia*** aff. *muriae* |  |  | X | X | X | X |  |
| *B* | *Wallemia canadensis* |  |  | X |  |  | X |  |
| *B* | *Wallemia* sp. |  |  |  | X |  | X |  |
| *A* | ***Zalaria*** *obscura* |  |  |  |  | X |  | NA+chx |

Legend: Species affiliation: Ascomycota (A), Basidiomycota (B); grey fields with X: species recorded on a single culture medium. NA=nutrient agar; M9= Minimal Medium; chx= cycloheximide.

## Supplementary Table 6. Attribute importance as identified by machine learning from data on material & sampling sites in relation to on-site detected fungi.

| **Attribute importance rank** | **Attribute group** | **Attribute name** | **Attribute score** |
| --- | --- | --- | --- |
| 1 | Organic painting constituents | proteins | 11.189 |
| 2 | Organic painting constituents | presumably tempera | 7.379 |
| 3 | Sampling sites | mouldy canvas | 5.789 |
| 4 | Sampling sites | mouldy paint layer (not edge) | 4.960 |
| 5 | Organic painting constituents | drying oil | 3.673 |
| 6 | Organic painting constituents | wax | 2.609 |
| 7 | Organic painting constituents | presumably drying oil | 2.387 |
| 8 | Organic painting constituents | presumably natural resin | 2.269 |
| 9 | Organic painting constituents | natural resin varnish | 2.265 |
| 10 | Organic painting constituents | starch | 1.864 |
| 11 | Organic painting constituents | presumably greasy tempera | 1.661 |
| 12 | Sampling sites | mouldy spider nets (back) | 0.973 |
| 13 | Sampling sites | mouldy glued canvas (back) | 0.854 |
| 14 | Organic painting constituents | greasy tempera | 0.628 |
| 15 | Sampling sites | mouldy paint layer (edge) | 0.433 |
| 16 | Organic painting constituents | oil or tempera | 0.103 |

## Supplementary Table 7. Attribute importance as identified by machine learning from data on painting material composition & sampling sites in relation to isolated fungi.

| **Attribute importance rank** | **Attribute group** | **Attribute name** | **Attribute score** |
| --- | --- | --- | --- |
| 1 | Organic painting constituents | presumably drying oil | 0.108 |
| 2 | Sampling sites | mouldy paint layer (not edge) | 0.081 |
| 3 | Organic painting constituents | presumably tempera | 0.074 |
| 4 | Organic painting constituents | proteins | 0.063 |
| 5 | Organic painting constituents | natural resin varnish | 0.058 |
| 6 | Sampling sites | mouldy canvas | 0.057 |
| 7 | Organic painting constituents | wax | 0.048 |
| 8 | Organic painting constituents | drying oil | 0.033 |
| 9 | Organic painting constituents | presumably greasy tempera | 0.019 |
| 10 | Organic painting constituents | greasy tempera | 0.016 |
| 11 | Sampling sites | mouldy glued canvas (back) | 0.015 |
| 12 | Sampling sites | mouldy paint layer (edge) | 0.013 |
| 13 | Sampling sites | mouldy spider nets (back) | 0.012 |
| 14 | Organic painting constituents | presumably natural resin | 0.009 |
| 15 | Organic painting constituents | starch | 0.001 |
| 16 | Organic painting constituents | oil or tempera | 0.000 |

## Supplementary Table 8. Attribute importance as identified by machine learning from data on observed damages and microscopically detected fungi.

| **Attribute importance rank** | **Attribute group** | **Attribute name** | **Attribute score** |
| --- | --- | --- | --- |
| 1 | Damage | paint flaking | 7.624 |
| 2 | Damage | grey stain | 6.508 |
| 3 | Damage | white stain | 5.942 |
| 4 | Damage | growth in paint cracks | 3.516 |
| 5 | Damage | paint cracking | 2.855 |
| 6 | Damage | moisture indicating stains | 1.473 |
| 7 | Damage | green stain | 0.522 |
| 8 | Damage | tearing canvas | 0.096 |

## Supplementary Table 9. Attribute importance as identified by machine learning from data on observed damages on paintings in relation to cultivated fungi.

| **Attribute importance rank** | **Attribute group** | **Attribute name** | **Attribute score** |
| --- | --- | --- | --- |
| 1 | Damage | paint cracking | 0.093 |
| 2 | Damage | paint flaking | 0.077 |
| 3 | Damage | white stain | 0.072 |
| 4 | Damage | grey stain | 0.058 |
| 5 | Damage | growth in paint cracks | 0.043 |
| 6 | Damage | tearing canvas | 0.016 |
| 7 | Damage | green stain | 0.014 |
| 8 | Damage | moisture indicating stains | 0.009 |

## Supplementary Table 10. Attribute importance as identified by machine learning from data on observed materials and damages on paintings in relation to cultivated *Aspergillus* species.

| **Attribute importance rank** | **Attribute group** | **Attribute name** | **Attribute score** |
| --- | --- | --- | --- |
| 1 | Organic painting constituents | presumably greasy tempera | 0.252 |
| 2 | Organic painting constituents | proteins | 0.210 |
| 3 | Sampling sites | mouldy paint layer (not edge) | 0.183 |
| 4 | Organic painting constituents | presumably tempera | 0.169 |
| 5 | Organic painting constituents | wax | 0.167 |
| 6 | Damage | paint cracking | 0.150 |
| 7 | Damage | grey stain | 0.147 |
| 8 | Organic painting constituents | natural resin varnish | 0.132 |
| 9 | Damage | white stain | 0.126 |
| 10 | Sampling sites | mouldy canvas | 0.090 |
| 11 | Organic painting constituents | presumably drying oil | 0.070 |
| 12 | Sampling sites | mouldy spider webs (back) | 0.067 |
| 13 | Damage | growth in paint cracks | 0.067 |
| 14 | Organic painting constituents | presumably natural resin | 0.066 |
| 15 | Damage | green stain | 0.064 |
| 16 | Sampling sites | mouldy glued canvas (back) | 0.063 |
| 17 | Organic painting constituents | drying oil | 0.052 |
| 18 | Damage | paint flaking | 0.041 |
| 19 | Organic painting constituents | greasy tempera | 0.035 |
| 20 | Damage | tearing canvas | 0.034 |
| 21 | Damage | moisture indicating stains | 0.019 |
| 22 | Sampling sites | mouldy paint layer (edge) | 0.010 |
| 23 | Organic painting constituents | starch | 0.008 |
| 24 | Organic painting constituents | oil or tempera | 0.003 |

**Supplementary Figure 1.** The Predictive Clustering Tree (PCT) for hierarchical multi-label classification (HMLC), predicting the presence of fungal taxa, isolated from sampled paintings of known/unknown material composition. The decision process starts by separating paintings painted presumably with drying oil binder to drying oil and tempera paintings. Alternatively, presumably tempera attribute is used to determine the prediction path. Later, varnish, wax and proteins are used to predict fungal taxa. Moldiness, presence of presumable natural resins and greasy tempera are also used but are not as important as previously mentioned attributes.


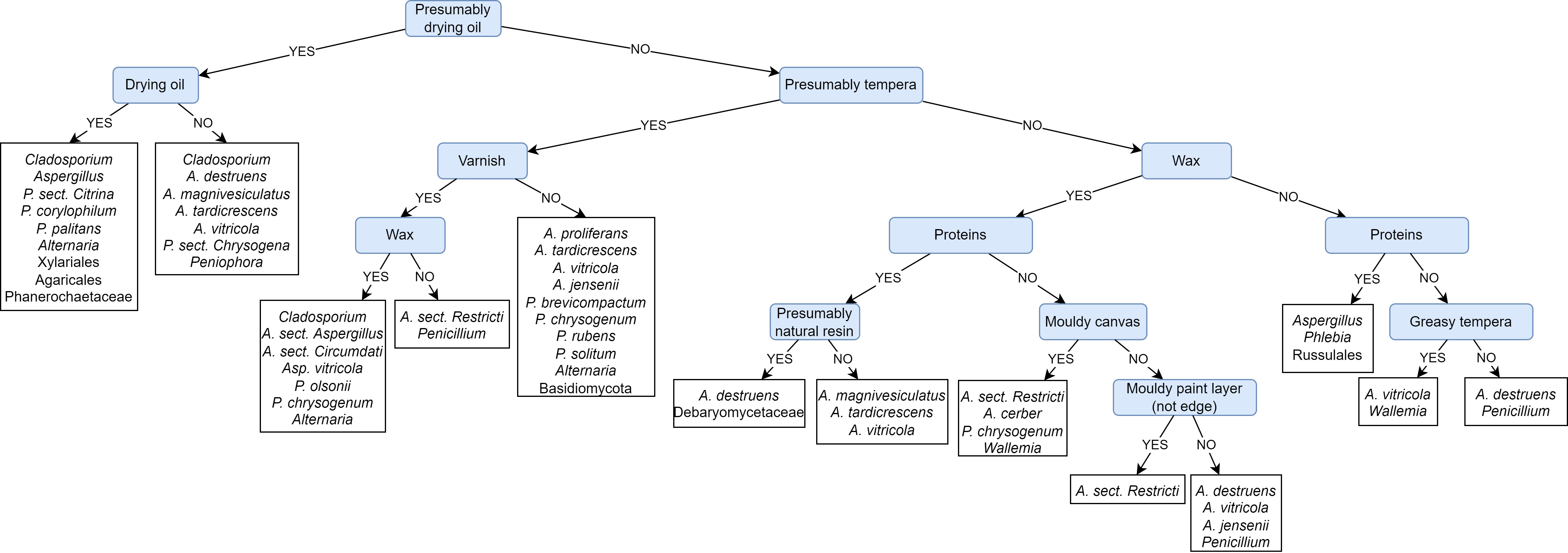


**Supplementary Figure 2.** The Predictive Clustering Tree (PCT) for multi-label classification (MLC) built on the data on observed materials and painting damages from associated cultivations of *Aspergillus* species. The decision process starts by separating paintings according to gray staining, and then depends on the presence of proteins (right side of tree) or presumably greasy tempera paints (left side).


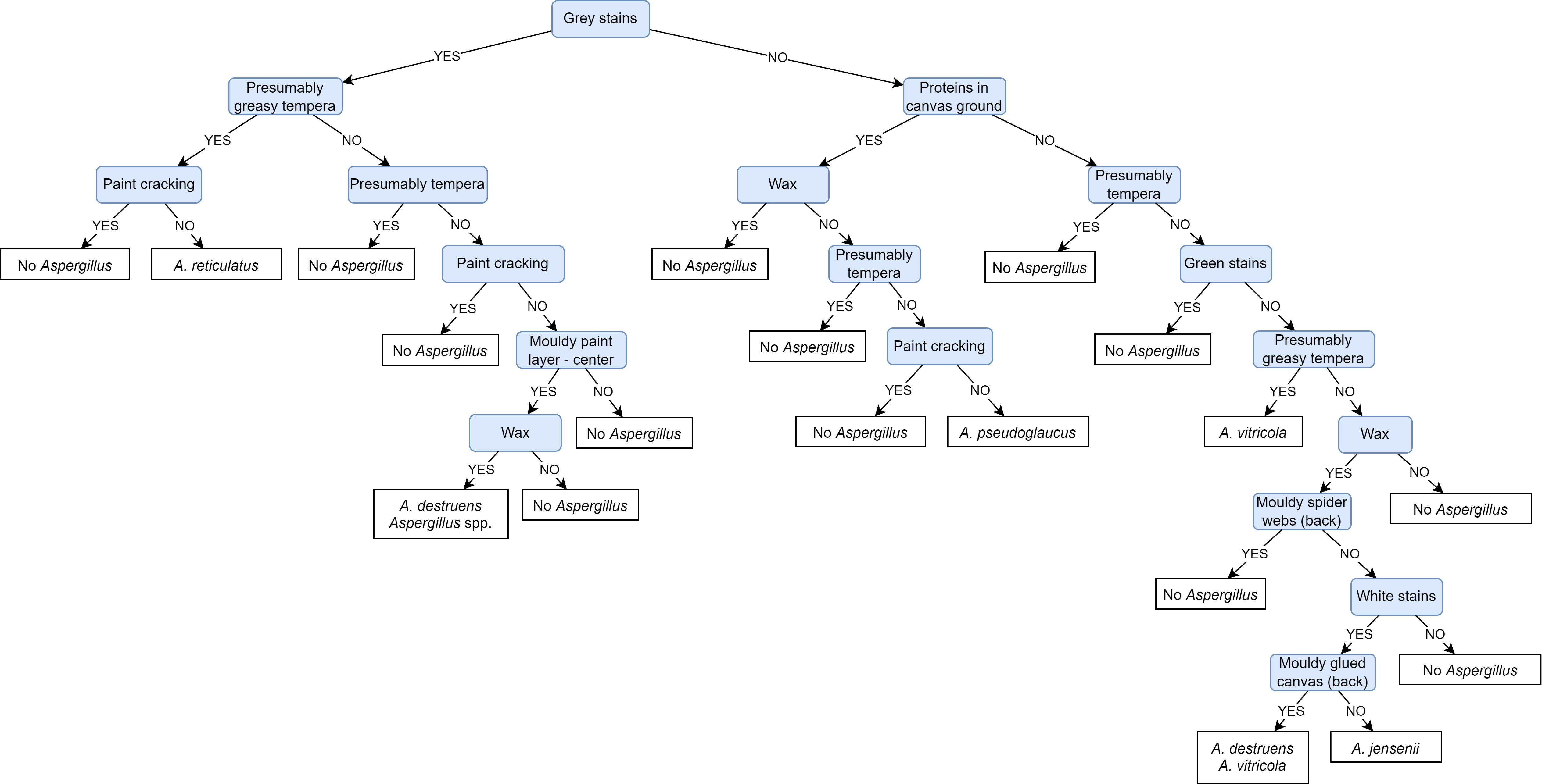

Supplement: Supplementary file 1 [file Data_Sheet_1.docx]
